# Single cell analysis reveals the roles and regulatory mechanisms of type-I interferons in Parkinson’s disease

**DOI:** 10.1186/s12964-024-01590-1

**Published:** 2024-04-02

**Authors:** Pusheng Quan, Xueying Li, Yao Si, Linlin Sun, Fei Fan Ding, Yuwei Fan, Han Liu, Chengqun Wei, Ruihua Li, Xue Zhao, Fan Yang, Lifen Yao

**Affiliations:** 1 Department of Neurology, The First Affiliated Hospital, Harbin Medical University, Harbin, China; 2 Department of Neurology, The Affiliated Hospital of Inner Mongolia Medical University, Hohhot, China; 3 Department of General Practice, Heilongjiang Provincial Hospital, Harbin, China

**Keywords:** Type-I interferons, Single cell, Immune, Transcription factors, Parkinson’s disease

## Abstract

The pathogenesis of Parkinson’s disease (PD) is strongly associated with neuroinflammation, and type I interferons (IFN-I) play a crucial role in regulating immune and inflammatory responses. However, the specific features of IFN in different cell types and the underlying mechanisms of PD have yet to be fully described. In this study, we analyzed the GSE157783 dataset, which includes 39,024 single-cell RNA sequencing results for five PD patients and six healthy controls from the Gene Expression Omnibus database. After cell type annotation, we intersected differentially expressed genes in each cell subcluster with genes collected in The Interferome database to generate an IFN-I-stimulated gene set (ISGs). Based on this gene set, we used the R package AUCell to score each cell, representing the IFN-I activity. Additionally, we performed monocle trajectory analysis, and single-cell regulatory network inference and clustering (SCENIC) to uncover the underlying mechanisms. In silico gene perturbation and subsequent experiments confirm NFATc2 regulation of type I interferon response and neuroinflammation. Our analysis revealed that microglia, endothelial cells, and pericytes exhibited the highest activity of IFN-I. Furthermore, single-cell trajectory detection demonstrated that microglia in the midbrain of PD patients were in a pro-inflammatory activation state, which was validated in the 1-Methyl-4-phenyl-1,2,3,6-tetrahydropyridine (MPTP)-induced PD mouse model as well. We identified transcription factors NFATc2, which was significantly up-regulated and involved in the expression of ISGs and activation of microglia in PD. In the 1-Methyl-4-phenylpyridinium (MPP^+^)-induced BV2 cell model, the suppression of NFATc2 resulted in a reduction in IFN-β levels, impeding the phosphorylation of STAT1, and attenuating the activation of the NF-κB pathway. Furthermore, the downregulation of NFATc2 mitigated the detrimental effects on SH-SY5Y cells co-cultured in conditioned medium. Our study highlights the critical role of microglia in type I interferon responses in PD. Additionally, we identified transcription factors NFATc2 as key regulators of aberrant type I interferon responses and microglial pro-inflammatory activation in PD. These findings provide new insights into the pathogenesis of PD and may have implications for the development of novel therapeutic strategies.

**Supplementary Information:**

The online version contains supplementary material available at 10.1186/s12964-024-01590-1.

## Introduction

As one of the fastest-growing neurological diseases, Parkinson’s disease (PD) is the second most common neurodegenerative disease associated with aging [1, 2]. It manifests as nonmotor (cognitive impairment, mood impairment, olfaction disturbances and autonomic dysfunction) and motor symptoms [3]. Symptomatic treatments are available, but none of them effectively slows or halts disease progression.

The etiology of PD is multifactorial and involves environmental factors, neuroinflammation, and genetic susceptibility [4]. Indeed, increasing evidence points to a causal role for inflammation in PD [5]. An aberrant immune response may arise in response to systemic viral infection, circulating IFNα, exotoxins, or accumulation of insoluble protein fibrils (e.g., α -synuclein, α-Syn) [6], and type I interferons (IFN-I) have been implicated as pivotal mediators in immune responses, with elevated levels observed in patients with Parkinson’s Disease (PD), potentially leading to neurotoxic effects [7–9]. Conversely, some literature suggests a neuroprotective role of IFN-I in PD, citing its involvement in modulating immune responses and potentially slowing disease progression [10].

IFNs are a family of proinflammatory and immunomodulatory cytokines with potent antiviral activities, the production of which can be attributed to a wide range of cell types, such as monocytes, macrophages, and microglia [11]. IFNs phosphorylate STAT1 and STAT2 when they engage in the heterodimeric IFN-α/β receptor (IFNAR). When IFNs bind to their receptors, they activate the JAK/STAT signaling pathway, which activates hundreds of IFN-I-stimulated genes (ISGs) [12]. Numerous nervous system pathologies are associated with IFN dysfunction, including PD, Alzheimer’s disease (AD), prion disease, and Huntington’s disease (HD) [13–15]. However, the expression features of ISGs in different cell types and their underlying mechanisms in PD have not been well described thus far.

In this study, we investigated the activity of IFN-I and the potential regulatory mechanisms underlying PD using single-cell RNA sequencing (scRNA) data and microarray transcriptomic profiles of human midbrain cells or tissues from patients with PD. In silico gene perturbation, the 1-Methyl-4-phenyl-1,2,3,6-tetrahydropyridine (MPTP)-induced PD mouse model and 1-Methyl-4-phenylpyridinium (MPP^+^) treated BV2 cells were used to validate the results.

## Methods

### Single-cell raw data quality control

From the Gene Expression Omnibus (GEO) database, we downloaded RNA sequencing data of midbrain specimens from five patients with idiopathic Parkinson’s disease and six healthy individuals (accession: GSE157783) [16]. Further analyses were performed using R (version 4.1.3) and the Seurat R package (version 4.1.1) [17]. For the initial QC step, cells with expression of < 200 or > 6000 genes and cells with < 200 or > 20,000 unique molecular identifiers (UMI) were filtered out of the analysis [18]. To integrate all 11 samples, we normalized the data using Seurat SCTransform for each sample and combined the samples following the Seurat CCA integration workflow. The top 3000 most highly variable genes were used for principal component analysis (PCA), then the first 20 principal components (PCs) were selected for downstream analysis.

### Cell type identification

An optimization algorithm based on the graph of shared-nearest neighbors (SNN) was used for unbiased clustering (FindClusters) based on the above 20 PCs (resolution = 2). We calculated the markers between each cluster of cells using the FindAllMarkers function, with a cutoff of |log 2 FC| ≥ 0.25, population expression ratio ≥ 0.25, and adjusted *p*-value < 0.05. Each cluster was manually assigned to one of the main cell types based on the expression of known marker genes. Then, cells were visualized using t-distributed Stochastic Neighbor Embedding (tSNE) plots. Meanwhile, Differential abundance testing was performed using miloR [19].

### IFN-I-stimulated genes score

To achieve reliable results, the Interferome database [20] was used to generate ISGs based on differentially expressed genes (|log 2 FC| > 5) in each cell subgroups, and the IFN-I-stimulated genes set was obtained to estimate the IFN-I activity of individual cells with the R package AUCell (version 1.12.0) [21]. The area under the curve (AUC) values were higher in cells expressing more genes within the gene set. To calculate the threshold for considering the current gene-set active, the function “AUCell_explore Thresholds” was used. Subsequently, cell clustering tSNE embeddings were colored according to AUC scores to display which the distribution of IFN-I scores in different cell subsets.

### Enrichment analysis

Gene Ontology (GO) [22] and Encyclopedia of Genes and Genome (KEGG) [23] pathway analysis for the differentially expressed genes (DEGs) in high-ISG-scoring cells were performed using Metascape (www.metascape.org), and then plotted using ggplot in R.

### Pseudotime analysis for IFN-I-related cell subsets

Pseudotime trajectories for the main cell types were constructed using the Monocle package (version 2.22.0) according to the operation manual [24]. Based on the highly variable genes identified by Monocle, we reduced the dimensions of the cells by using the DDRTree approach and reduceDimension function. Finally, cells were ordered along the pseudo time trajectory with orderCells, and the plot_cell_trajectory function was used for visualization. We generated a split heatmap of selected genes with significant changes over pseudotime, high differential expression, or known biologic identity through the Monocle function plot_genes_ branched_heatmap at branch_point 1 for microglia.

### In silico gene perturbation

In the context of deep learning, models incorporating context-awareness and attention mechanisms demonstrate the capability to make predictions in biologically constrained network environments with limited data. This accelerates the discovery of crucial network regulatory factors and candidate therapeutic targets. In this study, we employed the Geneformer tool to investigate key genes associated with the transition of microglia with high IFN-I scores to those with low IFN-I scores within the PD group [25–27].

### Single-cell regulatory network analysis

In order to identify the key transcription factors (TFs) in different cell types, the cis-regulatory analysis was performed using pySCENIC (version 0.11.2) [21, 28], a tool based on co-expression and DNA motif analysis to infer gene regulatory networks. Network activity was then assessed for each individual cell by calculating AUC. Briefly, we identified transcription factors using GENIE3, assembled them into modules (regulons), and analyzed them using RcisTarget with gene-motif rankings: 500 bp upstream and 100 bp downstream of the transcriptional start site (TSS). We next scored regulon activity for each cell in the data using AUCell. Finally, we plotted binarized regulon activity on the tSNE plots. TFs with adjusted by Benjamini–Hochberg false discovery rate < 0.05 were considered for further research. Next, the Pearson correlation coefficient was used to analyze the correlations between regulons and IFN-I scores.

### Transcriptomic data obtained and GSVA analysis

The GEO database was searched for bulk transcriptomics datasets containing the term “Parkinson’s disease” and the filter setting “*Homo sapiens*” to validate expression features of key TFs in substantia nigra in PD, and over 100 datasets related to PD were shown. Inclusion criteria were as follows: 1. Articles related to the dataset have been published; 2. Data have been made available for download; and 3. Tissues were extracted from Substantia nigra. The PCA was applied to identify and remove outlier samples. DEGs were calculated by the limma [29] (version 3.50.3) R package with an absolute log2 fold change > 0.25 and a *p* value of < 0.05, and then visualized with ggplot2. Afterwards, we used GSVA [30] (version 1.18.0) and to perform pathway enrichment between groups based on normalized count matrices, and pathways with a *p*-value less than 0.05 and a false discovery rate (FDR) less than 0.25 are considered to be differentially expressed between groups.

### Administration of MPTP to mice

Male C57BLmice (6–8 weeks old) were purchased from Vital River Laboratory Animal Technology Co., Ltd. Animals were housed in clean polypropylene cages under constant temperature and humidity, with an alternating 12-hour day-night cycle, during which they had free access to food and water. Mice were administered with MPTP (30 mg/kg, ip) daily for 5 days, and were sacrificed 7 days after last MPTP dose. Afterward, the midbrains of mice were obtained for further study. Animals were handled in accordance with Harbin Medical University’s Institutional Animal Care and Use Committee guidelines.

### Cell culture and model construction

Human neuroblastoma SH-SY5Y cells and BV2 microglial cells, extensively employed in Parkinson’s disease research, were cultured in DMEM (C11995500BT, Gibco, USA) supplemented with 10% FBS, 100 units of penicillin, and 100 μg/ml streptomycin. The cells were maintained at 37 °C in a 5% CO2 incubator. BV2 cells were cultured in media with or without MPP ^+^ (0.5 mM) for 24 h [31]. BV2 cells were divided into four groups: control group (CON), MPP^+^ group, si-NC+ MPP^+^, and si-NFATc2 + MPP^+^. We collected conditioned medium (CM) for four groups of cells separately and added them to SH-SY5Y for further co-culture for another 24 h. Subsequently, we measured the apoptosis level of SH-SY5Y. SiRNA sequences are shown in Additional file 2: Table S1.

### Immunofluorescence staining

Immunofluorescence staining was performed on 5-μm paraffin sections following standard protocols. Briefly, we incubated brain sections with primary antibodies overnight at 4 °C. The primary antibodies used were mouse anti-Iba1 (ab283319, 1: 100, Abcam, Cambridge, United Kingdom) and rabbit anti-Tyrosine Hydroxylase (ab6211, 1:1000, Abcam, Cambridge, United Kingdom). The sections were then washed with PBS and incubated for 1 h at 37 °C with fluorescein (FITC) or Texas Red-labeled secondary antibodies. Tissues were mounted onto slides, and 4′6′-diamidino-2′-phenylindole (DAPI) was used as a counterstain. Finally, images were acquired using a confocal laser scanning microscope (Leica, Wetzlar, Germany).

### Western blotting

The BV2 or SH-SY5Y cells or midbrain tissue lysates were subjected to Western blotting to detect NFATc2, STAT1, p-STAT1, IRF9, BAX, BCL-2, p65, p-p65, IκBα, p-IκBα and GAPDH. The following antibodies were used for protein detection by western blotting. NFATc2 (22023–1-AP, 1:1000, Proteintech), STAT1 (66545–1-lg, 1:2000, Proteintech), p-STAT1 (ab109461, 1:1000, Abcam), IRF9 (14167–1-AP, 1:1000, proteintech), BAX (A19684, 1:1000, ABclonal), BCL-2 (26593–1-AP, 1:1000, Proteintech), NF-kB p65 (AF5006, 1:1000, Affinity), Phospho-NF-kB p65(Ser536) (AF2006, 1:1000, Affinity), IKB alpha (AF5002, 1:1000, Affinity), Phospho-IKB alpha (Ser32/Ser36) (AF2002, 1:1000, Affinity), and GAPDH (#5174, 1:1000, Cell Signaling Technology).

### Measurement of cytokines and IFN-α/ IFN-β

The cell culture supernatants were collected according to the recommended kit protocol. The concentrations of IL-1β, IL-6, and TNF-α among different groups were quantified using ELISA. Additionally, the levels of IFN-α and IFN-β were concurrently assessed. The ELISA kits used were as follows: IL-1β (SEKM-0002), IL-6 (SEKM-0007), TNF-α (SEKM-0034) from Solaibao Biological Technology Co., Ltd., and IFN-α (JL12034-96 T), IFN-β (JL20219-96 T) from Jianglai Bio.

### Statistical analyses

Data are expressed as mean ± standard deviation (SD). In comparisons between two groups, Student’s t-tests were used to assess statistical significance, while comparisons between multiple groups were done using one- or two-way ANOVA followed by Benjamini–Hochberg correction. All images shown are representative results from a minimum of three independent biological replicates with similar trend. *P* values < 0.05 were considered to indicate statistical significance.

## Results

**Fig. 1:**
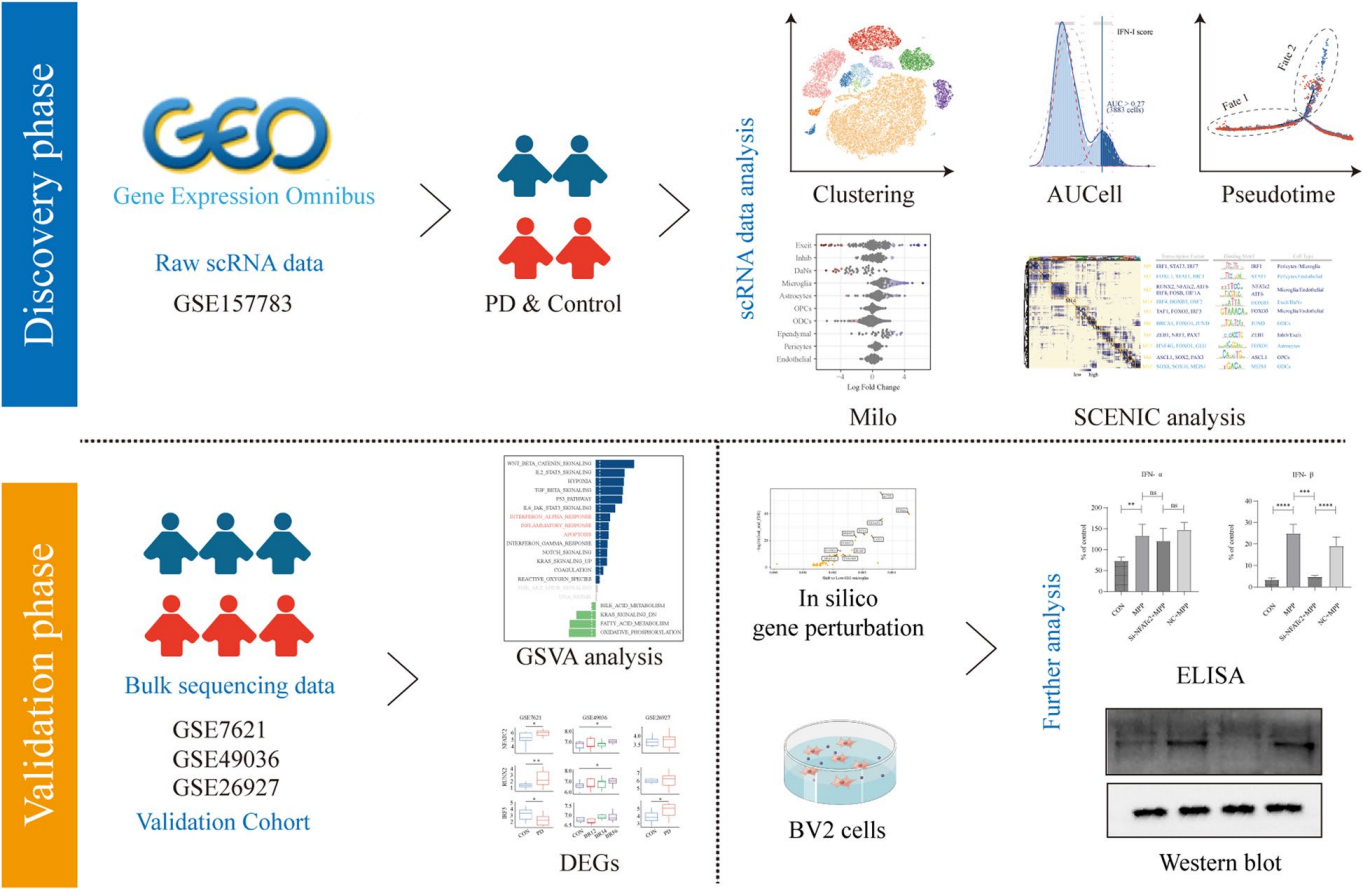
The workflow of this study

An overview of the study can be found in Fig. 1.

### scRNA data revealed the complexity of PD midbrain cells

**Fig. 2:**
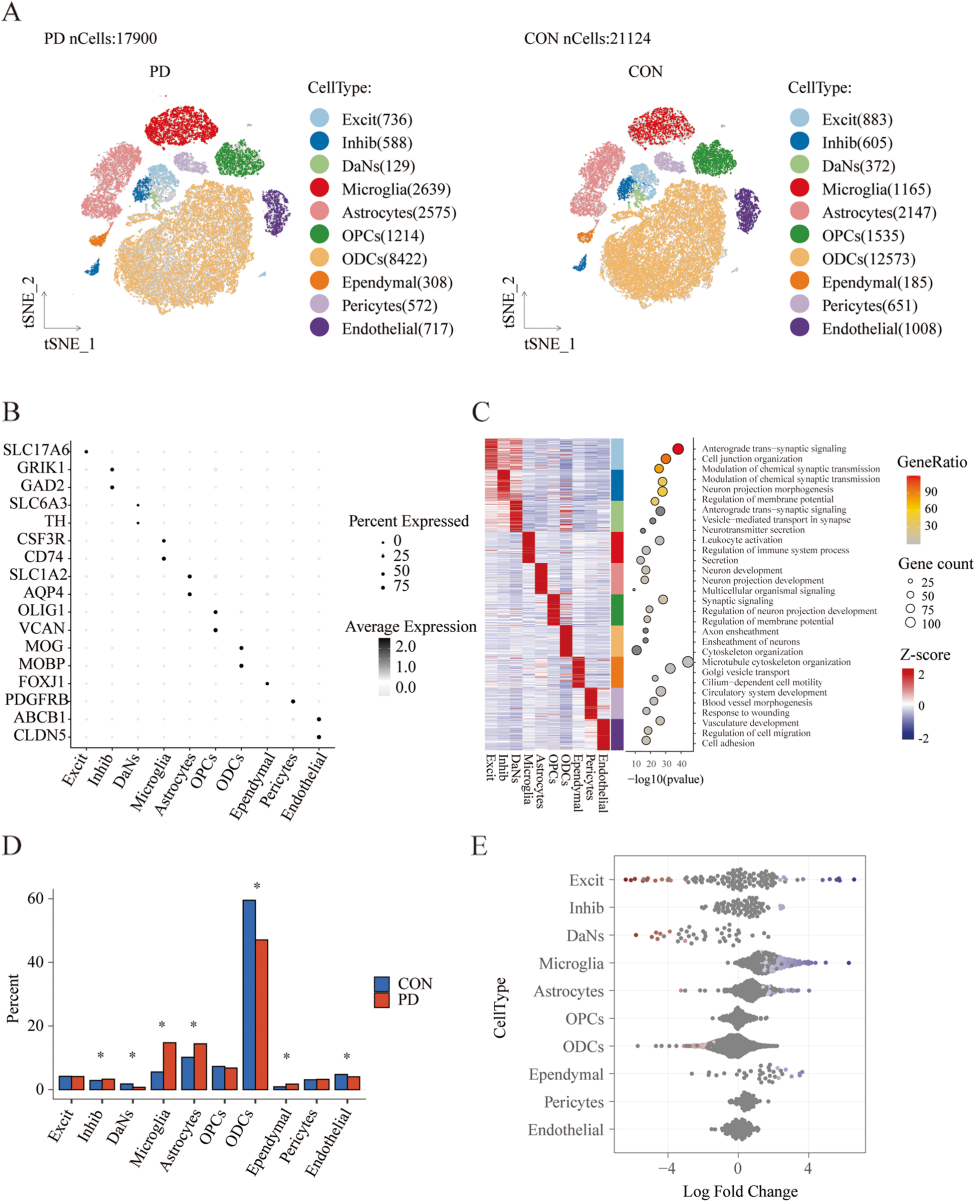
Single-cell transcriptome analysis of PD midbrain cells. **A** The tSNE plot representing the 10 cell types in different groups. **B** Dotplot showing average scRNA-seq expression of marker genes in different subgroups. **C** Heatmap showing expression of the top 50 DEGs in each cell subset and GO enrichment analysis results. **D** Bar plots showing the proportion of cell types in different groups. **E** MiloR exhibited a significant distinction, primarily characterized by a higher relative abundance of microglia observed in patients with PD

We analyzed the scRNA data of midbrain cells from six healthy controls and five PD patients. After data quality control, 39,024 single nuclei were retained for further analyses, with 21,124 and 17,900 in the control and PD groups, respectively (Additional file 1: Fig. S1A, B, and Additional file 2: Table S2). After normalization and PCA, we identified 32 clusters using the graph-based clustering, and visualized the clusters with tSNE plots (Additional file 1: Fig. S1C). These clusters were then annotated to known biological cell types based on canonical marker genes from the literature. Ultimately, we identified 10 cell subsets (Fig. 2A, B). Additionally, we identified highly expressed genes in each cell subset and performed GO analysis on them (Fig. 2C). Oligodendrocytes (ODCs) were marked by the expression of MOBP and MOG, and the differently expressed genes were enriched in axon ensheathment, ensheathment of neurons, cytoskeleton organization, etc., well supporting its identity. Microglia were annotated with CD74 and CSF3R, and their genes were enriched in leukocyte activation, regulation of immune system process, secretion, etc. Cells expressing SLC17A6 and either GAD2 or GRIK1 were classified as excitatory (Excit) and inhibitory neurons (Inhib). Dopaminergic neurons (DaNs) were characterized with TH and SLC6A3, and the differently expressed genes were enriched for anterograde trans-synaptic signaling, vesicle-mediated transport in synapse, and neurotransmitter secretion. Besides, oligodendrocyte precursor cells (OPCs) were characterized by the expression of VCAN and OLIG1, astrocytes by the expression of AQP4, and ependymal cells by the expression of FOXJ1. Regarding vascular cells, endothelial cells were identified by CLDN5, and pericytes by PDGFRB. Cell subset proportions varied greatly among individuals (Additional file 1: Fig. S1D). Altogether, these results demonstrated an increase in microglia and astrocytes, as well as a decrease in oligodendrocytes and DaNs in the midbrains of idiopathic PD patients (Fig. 2D). We applied the miloR tool to quantify shifts in the abundance of all cell types between groups, and the most noticeable difference was the greater relative abundance of microglia present in the PD patients (Fig. 2E).

### Microglial exhibit higher IFN-I activity

**Fig. 3:**
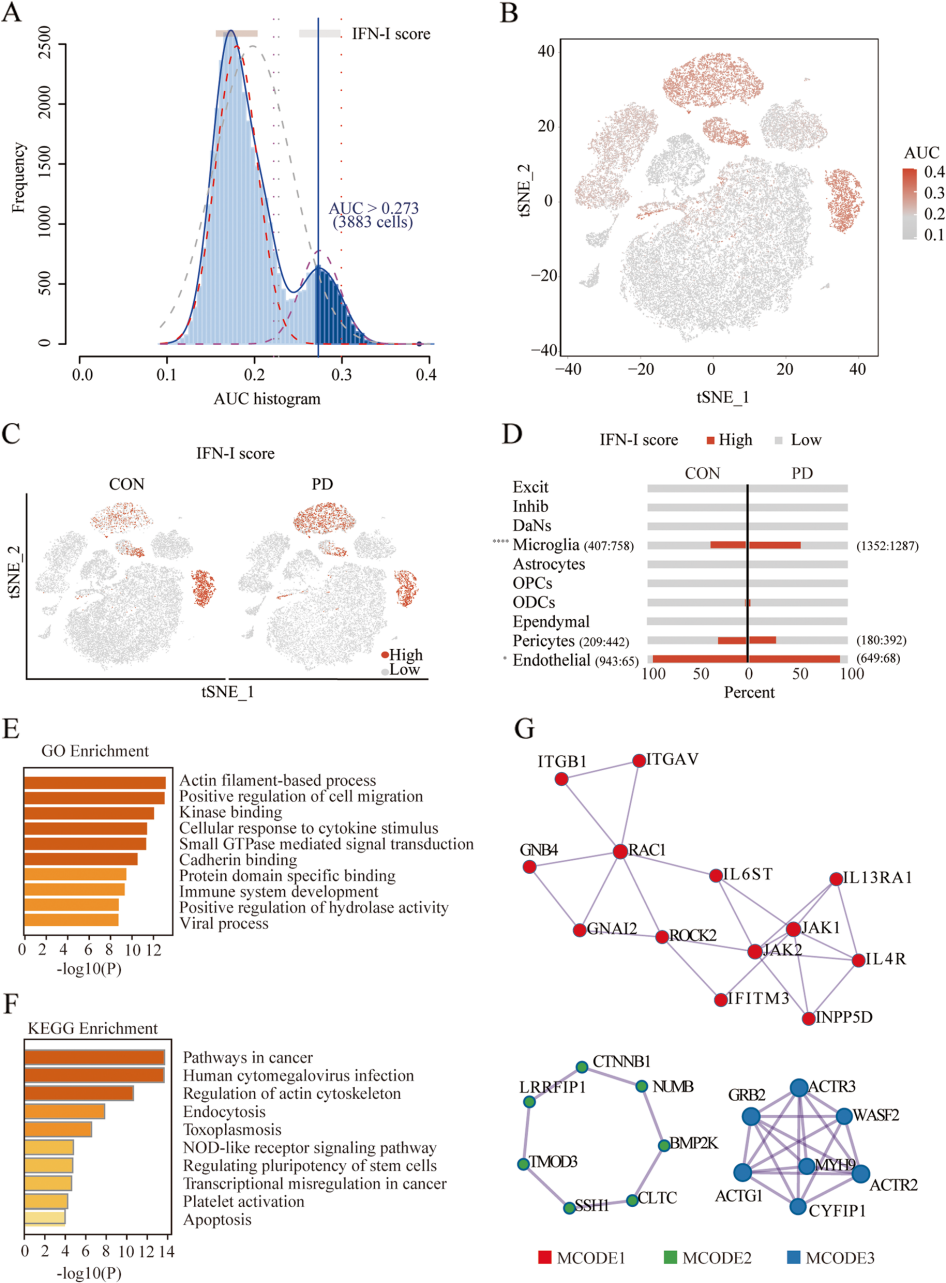
IFN-I scores for PD midbrain cell subsets. **A** An IFN-I score was calculated based on 469 screened ISGs. The threshold value was 0.273, and 3883 cells exceeded it. **B** The tSNE plot of each cell based on the IFN-I score. High-IFN-I-scoring cells are highlighted with red color. **C** The tSNE plot based on the IFN-I scores between different groups. **D** The bar plot showing the percent of High-IFN-I-scoring cells in each cell subset between groups. Of 2639 microglial cells in the PD group, 1352 showed high IFN-I activity, contrasting with 407 of 1165 cells in the control group. For endothelial cells, 649 of 717 in the PD group had high IFN-I activity versus 943 of 1008 in the control group. However, comparing pericytes—180 high IFN-I activity of 572 in the PD group against 209 of 651 in the control. **E, F** GO and KEGG enrichment analysis of DEGs in high-IFN-I-scoring cells. **G** PPI analysis (MCODE) of DEGs in high-IFN-I-scoring cells. *p < 0.05; ****p < 0.0001

The differentially expressed genes (logFC > 5) were imported into the Interferome database, and a 469-ISGs gene set was obtained to estimate the IFN-I activity of individual cells (Additional file 2: Table S3). The AUC values of all cells exhibited two peaks, with 3883 cells displaying comparatively higher AUC values with an AUC threshold of 0.273 (Fig. 3A). The high-scoring cells were mainly microglia, endothelial, and pericytes (Fig. 3B). More microglia exhibited high-IFN-I activity in the PD group compared with the control group (Fig. 3C, D). Specifically, in the PD cohort, 1352 out of 2639 microglial cells exhibited high IFN-I activity, a significant contrast to the control group, where only 407 out of 1165 cells displayed similar activity levels, resulting in a statistically significant disparity (*p* = 2.111e-20). Additionally, among endothelial cells, a significant difference was observed: 649 out of 717 cells in the PD group showed high IFN-I activity compared to 943 out of 1008 in the control group (*p* = 0.0252). However, a further analysis of pericytes revealed no significant difference, with 180 out of 572 cells in the PD group and 209 out of 651 in the control group showing high IFN-I activity (*p* = 0.8597). Enrichment for KEGG and GO gene sets were assessed separately to explore functional characteristics of these high-scoring cells. We found that they were mainly related to cellular response to cytokine stimulus, cell migration, immune system development, infection, and apoptosis (Fig. 3E, F). In addition, three functional subnet modules (MCODE 1, 2, and 3) were selected from the PPI network. Notably, the core modulated genes (e.g., JAK1, JAK2, RAC1, IFITM3, etc.) were highly correlated with the IFN pathway (Fig. 3G).

### Inter-group differentiation of microglial shows distinct trends

**Fig. 4:**
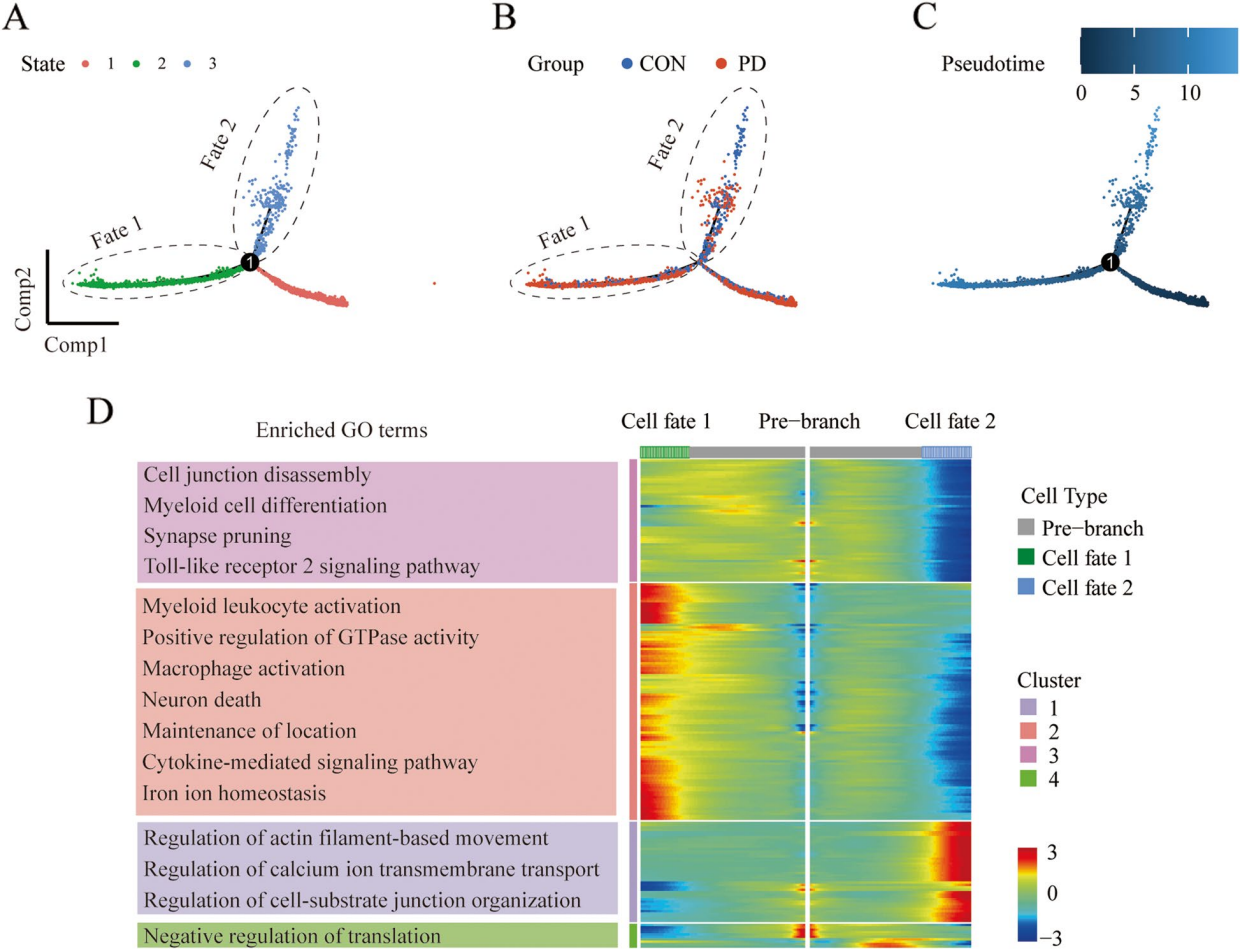
Pseudotime and single cell trajectory analysis for microglia by Monocle. **A** Three stages of microglia differentiation. State 1 is the earliest stage of differentiation. **B** Differentiation of microglia between groups. **C** Timing differences in cell differentiation. Darker blue indicates an earlier stage of differentiation while lighter blue indicates a later stage. **D** Clusters of genes that were differentially expressed across pseudotime at branch_point 1. The represented biological pathways from GO analysis of each cluster are noted at left

We used Monocle to order the cells in microglia, endothelial, and pericytes lineages along pseudotime and reconstruct lineage trajectories. State 1 was likely to be homeostatic on the entire microglial population (Fig. 4A-C). Microglia displayed a marked difference in cell differentiation between controls and PD patients at branch_point 1. Noticeably, state 2 (cell fate1) may represent a pro-inflammatory condition with expression of genes in cluster 2 (e.g., GPNMB, HSPA5, IL1RAP, etc.), and the embedded GO analysis revealed the “myeloid leukocyte activation”, and “neuron death” as the top enriched functional processes in cluster 2 (Fig. 4D), supporting the evidence linking microglia to both pro-inflammation and neuron loss. In contrast, there was no marked difference in cell differentiation for endothelial cells and pericytes (Additional file 1: Fig. S2).

### Toll-like receptor (TLR) family analysis

**Fig. 5:**
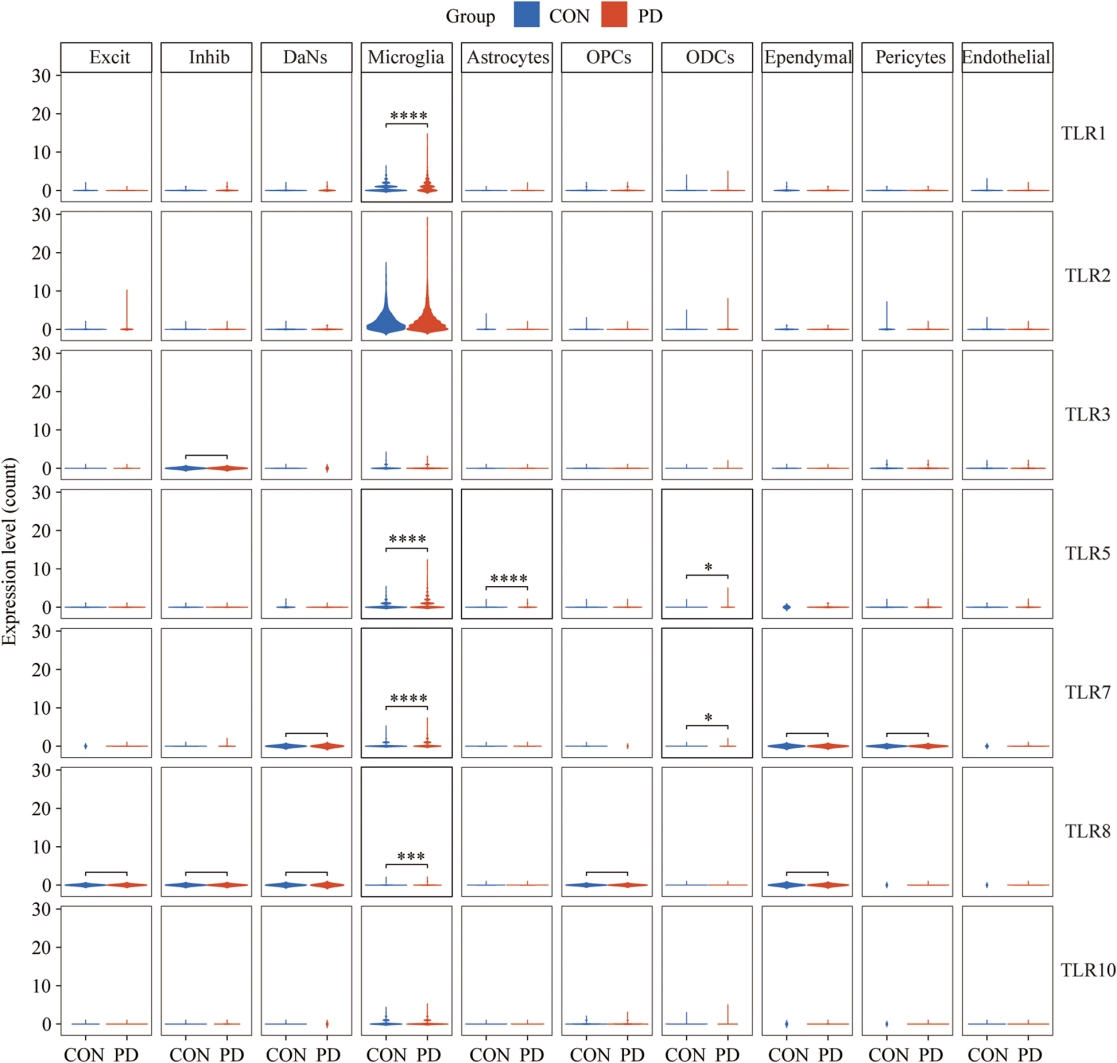
Violin plots showing TLR family expression in each cell type. *p < 0.05; **p < 0.01; ***p < 0.001, ****p < 0.0001

Due to the critical role that Toll-like receptors play in innate immunity and inflammation, the expression of the TLR family was displayed to investigate which were associated with disease pathogenesis. Totally, TLR1, TLR5, TLR7, and TLR8 greatly increased in PD, which were mainly located in microglia (Fig. 5). It appears that multiple TLR-dependent pathways may be involved in regulating IFNs production.

### NFATc2 serves as a key transcription factor in the phenotypic transition of microglial

**Fig. 6:**
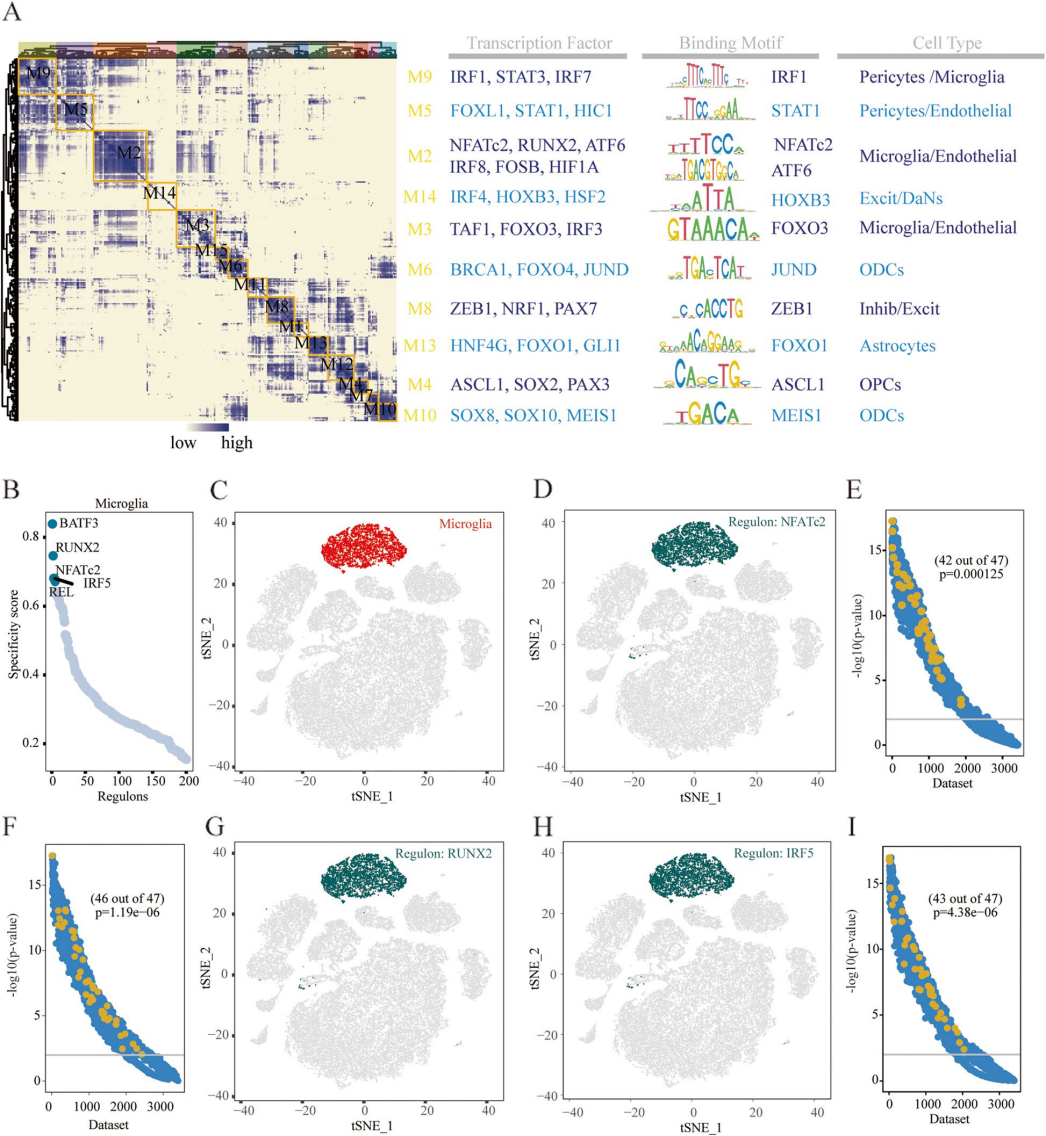
Identification of combinatorial regulon modules and Cell-type-specific regulon activity analysis. **A** Combinatorial regulation modules are identified based on regulatory connection specificity index matrices, along with representative transcription factors, corresponding binding motifs, and associated cell types. **B** Rank for regulons in Microglia based on regulon specificity score (RSS). **C** The t-SNE map highlights microglia (red dots). **D** A t-SNE map with binarized regulon activity scores (RAS) for top regulon NFATc2 (dark green dots). **E** Seek co-expression results for top regulon NFATc2 target genes in different GEO datasets. Each dataset is represented by an x axis, and its co-expression significance is represented by each y axis. Microglia related datasets with significant correlation (*p*-value < 0.01) are highlighted by yellow dots. **F, G** Same as E, D but for RUNX2. **H, I** Same as D, E but for IRF5

Next, we performed SCENIC analysis to identify the key transcription factors responsible for cell state transitions during disease development, which revealed distinct gene regulatory networks (GRNs, termed regulons) in distinct cells. A GRN is a set of transcription factors and cofactors which determines downstream gene expression and results in specific cell states. After preliminary analyses, we identified 196 out of 292 regulons that were predicted to be active in PD. To further analyze the TFs predicted to be active within microglia, we imputed all regulons using an entropic strategy to re-cluster all regulons identified above. The dot plots and heatmap were used to characterize the transcriptional activity of the M1–M16 modules (Fig. 6A). We found microglia were significantly enriched in the M2 and M3 modules, in which, 33 regulons were mostly specific to the high IFN-I-scoring microglia and PD (Additional file 1: Fig. S3, and Fig. S4A).

To further narrow down the list of potential candidate genes, we employed a strategy where the top five TFs across various cell types were meticulously ranked according to their regulon specificity scores (RSS). This analytical approach revealed that RUNX2, NFATc2, and IRF5 emerged as the regulons with the highest specificity, closely associated with microglia exhibiting high IFN-I scores. This decision was informed by the observation that BATF3 and REL did not feature among the previously identified list of 33 regulons, guiding our selection towards those with a more direct relevance to our study’s focus (Fig. 6B**,** and Additional file 1: Fig. S4A). The tSNE plot provides additional evidence that these regulons are highly specific to microglia (Fig. 6C, D, G, H). To validate whether genes in the three regulons co-express significantly, we used SEEK analyzed of GEO datasets. The microglia-related datasets ranked highly out of more than 3000 datasets examined by SEEK (Fig. 6E, F, I). To confirm this difference using another method, the correlations between these three regulons and IFN-I scores in microglia in PD group were assessed using Pearson correlation coefficients (Additional file 1: Fig. S4C-E).

**Fig. 7:**
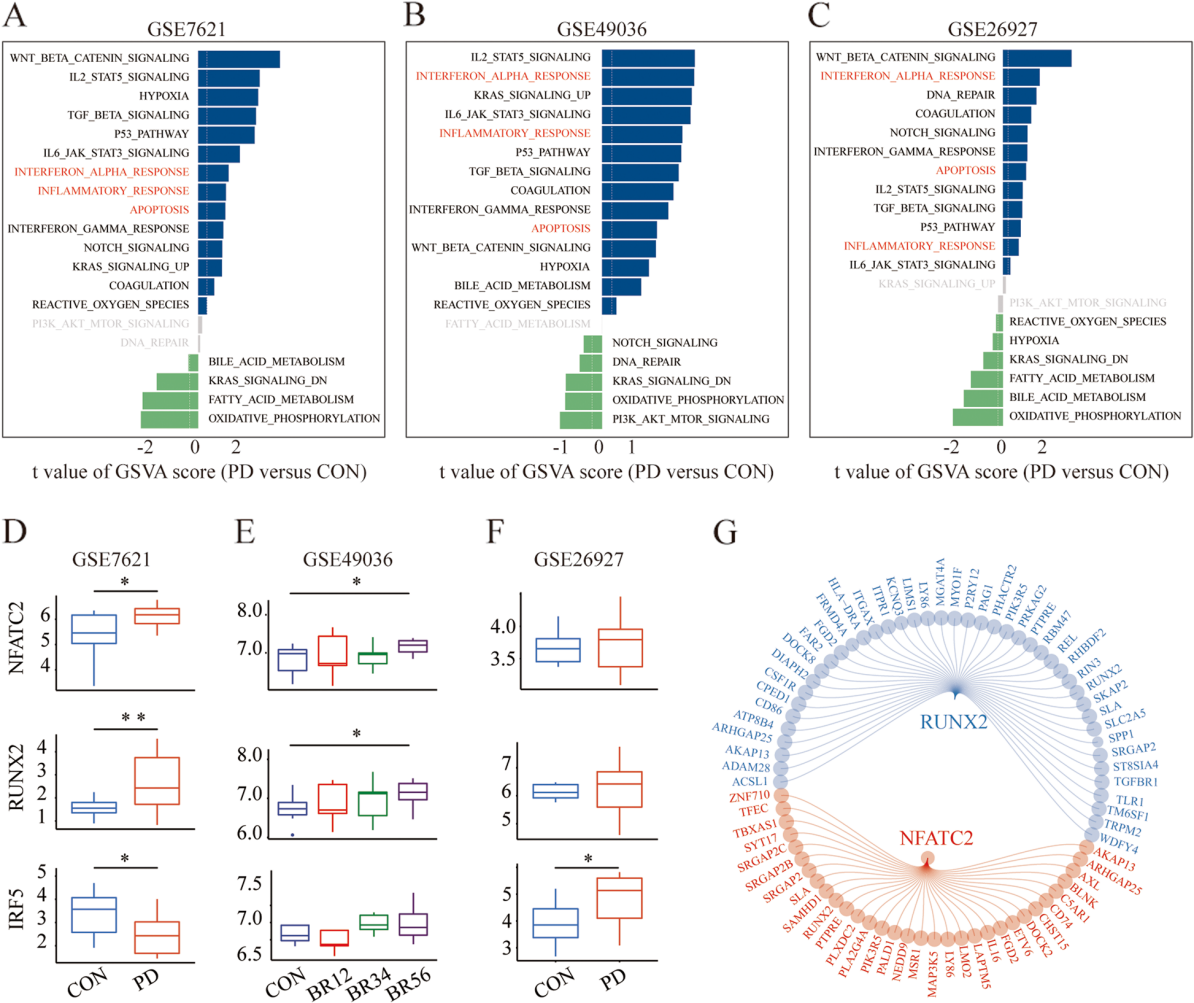
GSVA analysis and gene expression compare. **A-C** GSVA analysis for genes in GSE7621, GSE49036, and GSE26927, respectively. Red font represents the pathways exhibiting significant changes in different groups (*p* < 0.05 & FDR < 0.25). **D-F** Expression of NFATc2, RUNX2, and IRF5 in GSE7621, GSE49036, and GSE26927, respectively. **G** The regulatory network of NFATc2 and RUNX2

Raw data of GSE7621 [32], GSE99036 [33], and GSE26927 [34] were obtained from the GEO database, and one outlier identified by PCA was excluded (Additional file 1: Fig. S5). GSVA analyses were conducted to reveal the functional characteristics of all genes and the pathway alterations in PD. Unsurprisingly, we did identify differences in substantia nigra tissues, revealing PD-associated increases in interferon response, inflammatory response, and apoptosis (Fig. 7A-C). The expression of the top 3 key TFs in the three GSE datasets was shown in Fig. 7D-F. Specifically, the levels of NFATc2 and RUNX2 were markedly elevated in PD (GSE7621) and Braak stages 5/6 (GSE49036) relative to the control subjects, but not in GSE26927. IRF5 exhibited a contrary trend in GSE7621 and GSE49036. The key TFs and their target genes were shown in Fig. 7G.

**Fig. 8:**
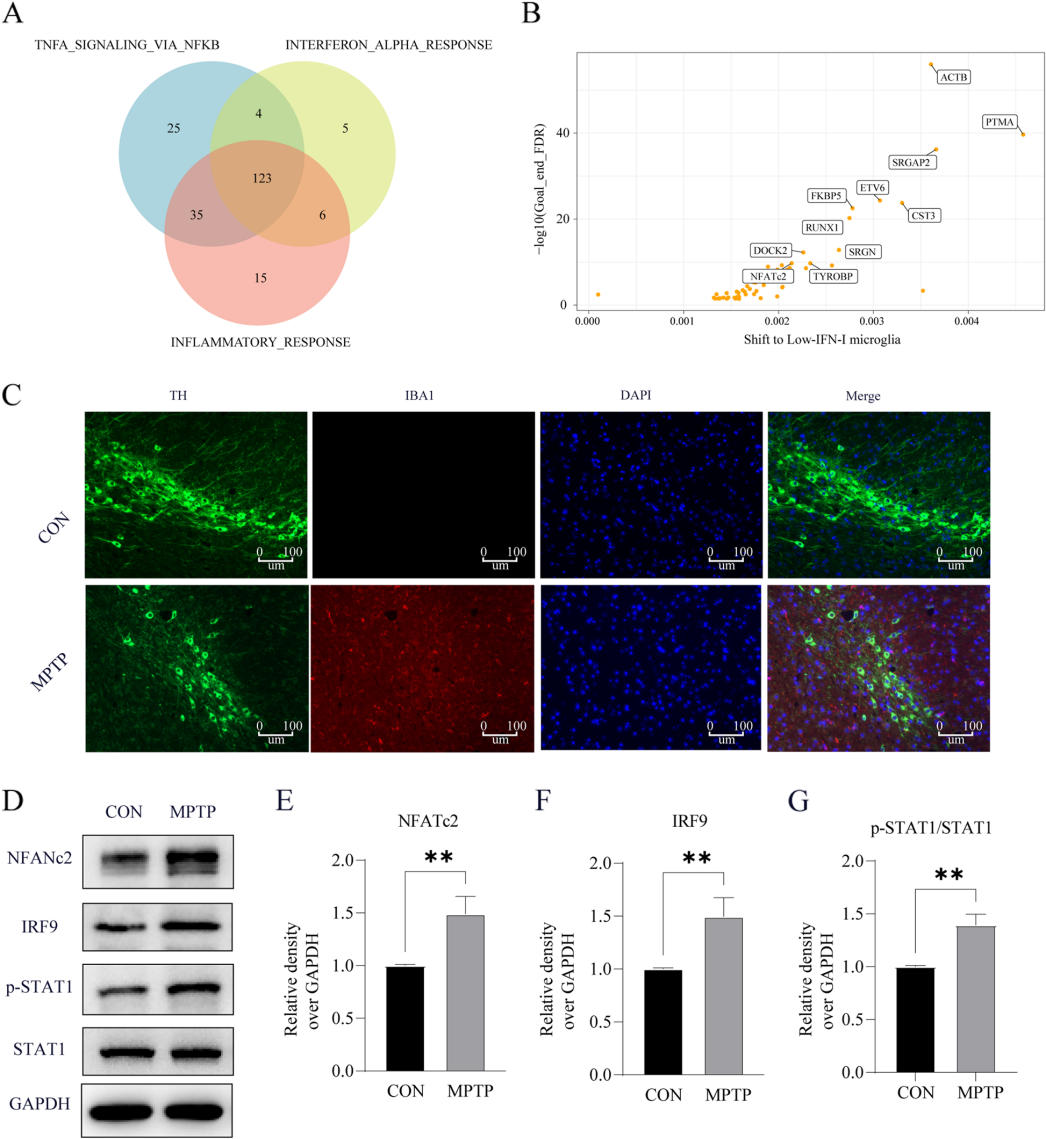
In silico gene perturbation and in vivo validation for NFATc2. **A** Venn diagram shows the overlap of transcription factors regulating the NF-κB, IFN-I response, and inflammatory response pathway. **B** The in silico gene perturbation results suggest key genes involved in the transition from high IFN-I microglial cells to low IFN-I microglial cells. **C** Iba1 show the increasing trend of microgliosis in MPTP-treated mice. **D-G** Western blot results for NFATc2 (**E**), IRF9 (**F**), and p-STAT1/ STAT1(**G**)

At the single-cell level, we enriched regulons involved in the regulation of NF-κB, IFN-I response, and inflammatory response pathways. We discovered that among the 123 regulons collectively regulating these three pathways, NFATc2 is implicated (Fig. 8A, Additional file 2: Table S4). In silico gene perturbation, we identified a total of 72 genes playing crucial roles in the transition of microglial cells from high IFN-I scores to low IFN-I scores in PD (Fig. 8B**,** Additional file 3: Table S5). This includes genes such as ACTB, ETV6, RUNX1, FBKP5, and NFATC2, reaffirming the critical role of NFATc2. Based on the above analysis of the results, NFATc2 was considered for further validation.

### Increased activation of microglial in the PD mice

The above results suggest IFN-related cells, especially microglia, may largely increase in the midbrain during PD and contribute to the exacerbation of PD. Therefore, the MPTP- treated mice were used for validation. In the midbrains of four groups of mice, we stained microglia with Iba-1 to compare their number through immunostaining. Consistently, we observed significantly increased Iba-1 microglia in the midbrains from PD (Fig. 8C). Meanwhile, as shown in Fig. 8D-G**,** NFATc2, p-STAT1/STAT1 and IRF9 were upregulated in MTPT-treated mice. Taken together, these results indicate that type I interferon pathway in midbrain is critically involved in the pathogenesis of PD.

### NFATc2 enhances the release of pro-inflammatory factors and the IFN-I response in microglial

**Fig. 9:**
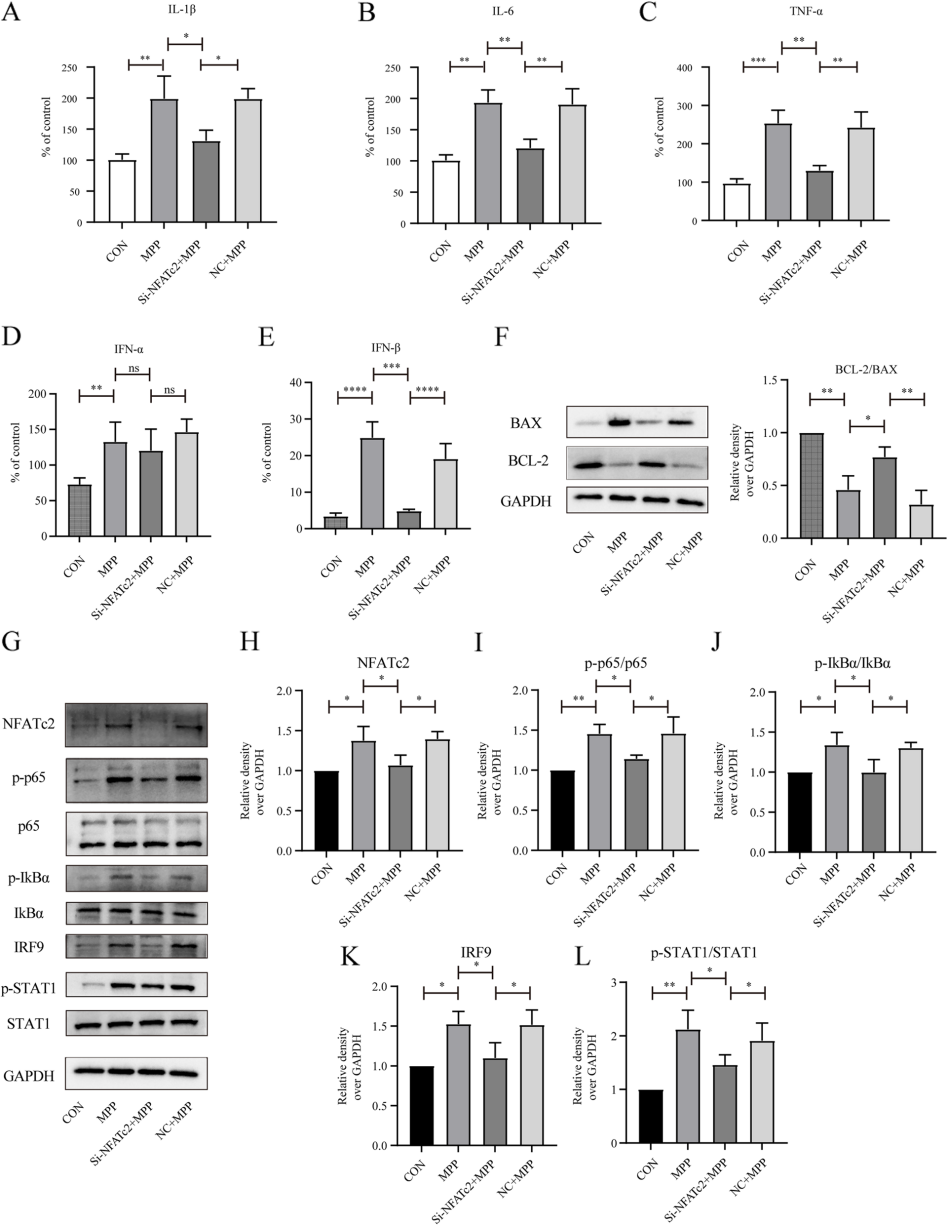
**A-C** ELISA results indicate that NFATc2 regulates the release of proinflammatory cytokines IL-1β (**A**), IL-6 (**B**), and TNF-α (**C**) in MPP^+^-induced BV-2 cells. **D-E** NFATc2 regulates the levels of IFN-β rather than IFN-α. **F** Knocking down NFATc2 can alleviate apoptosis in SH-SY5Y cells co-cultured in conditioned medium. **G-L** Western blot and the corresponding statistical analysis for NFATc2 (**H**), p-p65/ p65 (**I**), p-IκBα/IκBα(**J**), IRF9 (**K**), and p-STAT1/STAT1(**L**)

In our study, we aimed to explore the differences in Type I interferon (IFN-I) responses and neuroinflammation between Parkinson’s Disease (PD) patients and a control group. Our initial findings showed significant variations, leading us to investigate the role of NFATc2 in these processes. We measured critical pro-inflammatory cytokines, TNF-α, IL-1β and IL-6, involved in neurodegeneration and inflammation, and focused on key components of the IFN-I pathway, including STAT1, IFN-α, and IFN-β. We found that knockdown of NFATc2 significantly reduced the secretion of pro-inflammatory cytokines in MPP^+^-induced BV2 cells (Fig. 9A-C). Interestingly, NFATc2 regulates the levels of IFN-β in microglial cells rather than the levels of IFN-α (Fig. 9D, E). Additionally, knockdown of NFATc2 alleviated the damage to SH-SY5Y cells co-cultured in conditioned medium (Fig. 9F). In BV2 cells subjected to MPP^+^ treatment, a conspicuous activation of the NF-κB pathway was observed. Notably, the knockdown of NFATc2 was identified as an inhibitory factor against the activation of the NF-κB pathway (Fig. 9G-J). Moreover, our observations indicate that NFATc2 has the capacity to partially suppress the escalating expression trend of IRF9 and phosphorylated STAT1 induced by MPP^+^ (Fig. 9G, K, L).

## Discussion

Traditionally, PD was considered a movement disorder. However, the disease is now believed to be a multisystem disorder accompanied by neuroinflammation and immune dysfunction, which can manifest as non-motor symptoms in addition to motor symptoms. As is well established, uncontrolled immune cell infiltration into the central nervous system (CNS) in response to threats can have far more detrimental effects than the primary pathogenic conditions causing it. Given the crucial roles of IFNs in regulating immune and inflammatory responses, we quantified the features of ISGs and explored the underlying mechanisms using scRNA-seq. After quality control, 39,024 glial, neuronal, and vascular cells were used for analysis. Interestingly, we observed an increase in astrocytes and microglia, which coincided with a decrease in oligodendrocyte and DaNs fractions in the midbrain of individuals with idiopathic PD.

Interferons were considered to have pleiotropic functions through the regulation of cellular proliferation, death, and activation via triggering the expression of subsets of ISGs. Particularly, IFNs have been found to be active in PD [35, 36]. However, which factors prompted this change is unclear. Therefore, we assessed the ISG activity of the cell subgroups by calculating the IFN-I score for each cell. The high-scoring cells were mainly microglia, endothelial cells, and pericytes, especially microglia, in PD. As expected, an analysis of the GO and pathway enrichment of DEGs in these cells highlighted the cellular response to cytokine stimulus, cell migration, immune system development, infection, and apoptosis. Studies have found that many cell types, including neurons and glia, can produce IFN-I in the CNS [37]. Microglia, which reside in the central nervous system, are robustly responsive to IFN-I [38]. In addition, it appears that IFN-I have a positive effect on microglial phenotypes. Preliminary studies have shown that an overactivated IFNAR signal leads to inflammatory microgliosis in mouse models lacking ubiquitin-specific peptidase 18 [39], while blocking IFNAR signaling in aged mice attenuates the microglial pro-inflammatory phenotype in the CNS [40]. Consistent with these previous studies, we observed increased pro-inflammatory microglial infiltration in the PD midbrain. Additionally, both neurons and astrocytes have been shown to produce and respond to IFNs, but we did not observe significant ISG activation in these cells [41, 42]. Interestingly, some pericytes and endothelial cells scored high for IFN-I in physiological and pathological states, but their roles in PD development and IFN activation have not been fully elucidated. In summary, these results suggest that the activation of the IFN-I signaling pathway may involve multiple cell types in PD.

Pseudotime analysis in our study is utilized to infer the progression of cellular states within PD pathology, rather than tracking changes over actual chronological time. In our analysis, the ‘stages’ refer to hypothetical points along a continuum of disease progression or microglial activation states rather than temporal stages in development, as seen in neurogenesis studies. Regarding cell fates in the context of PD, we use the term to describe the potential functional states or roles that microglia might adopt in response to PD pathology. For example, early-stage fates might involve neuroprotective roles, whereas later stages might reflect a shift towards pro-inflammatory or neurodegenerative functions. This conceptual framework allows us to hypothesize about the dynamic roles of microglia in PD progression and to identify gene expression patterns that are indicative of these functional shifts.

The stimulation of TLRs by their ligands initiates the activation of intracellular signal transduction networks that drive the ensuing inflammatory response. Furthermore, TLRs are elevated in the brains of patients with PD [43]. In the present study, TLR1, TLR5, TLR7, and TLR8 were significantly elevated in patients with PD. Beraud et al. found that stimulation of murine BV2 microglial cells with α-Syn led to increased expression of TLR1 and TLR7 [44], which is consistent with our observations. Although no significant changes in the expression of TLR2 were found in this study, a trend toward an increase in PD was observed. It has been reported that pathological accumulation of α-Syn is positively correlated with increased TLR2 expression in the anterior cingulate cortex and striatum of human brains with PD [45]. Additionally, there are a small number of reports correlating TLR5 and TLR8 with PD [46, 47]. Unlike the aforementioned infectious diseases, TLRs and the initiation of neuroinflammation, including neurodegenerative disorders, can be triggered in the presence of toxic metabolites and proteins in many sterile CNS conditions. Despite this, TLR research is still in its infancy, and future studies should explore this topic in greater detail.

Distinct cell states and lineages are typically controlled by different variations in gene regulatory networks, and hence, cells might exhibit different phenotypes or functions. These findings suggest an important role of microglia in PD. Hence, we sought to identify the IFN-I regulatory factors associated with shaping microglial pro-inflammatory phenotypes. SCENIC analysis of microglia has successfully demonstrated the activity of known TFs, including NFATc2 and RUNX2. Geneformer is a pre-trained deep learning model that can be fine-tuned for a diverse array of downstream applications, expediting the identification of crucial network regulators and potential therapeutic targets [25]. Subsequent in silico gene perturbation analysis with Geneformer suggests a pivotal role of NFATc2 in the state transition of microglia in PD. A recent study demonstrated that NFATc2 is selectively phosphorylated and translocated into the nucleus via LRRK2 activation, resulting in neuroinflammatory activation [48]. However, another study suggested that LRRK2 modulates cytokine secretion in response to IFN-γ in an NFAT-independent manner in microglia. Moreover, spinal nerve ligation-induced pain can be attenuated by the global or microglia-specific deletion of Nfatc2 [49]. Heat shock transcription factor 1 (HSF1) downregulates NFATc2 expression, alleviating neuroinflammation, indicating a pro-inflammatory function of NFATc2 [50]. NFATc2 also plays a crucial role peripherally, as the loss of the deubiquitinating enzyme USP15 indirectly inhibits NFATc2 degradation, promoting T cell activation [51]. In this study, we delineate the role of NFATc2 as a facilitator in the upregulation of pro-inflammatory cytokines in microglial cells. Nevertheless, the relative roles of NFATc2 in IFN progression require further clarification.

Knockdown of NFATc2 resulted in a marked decrease in STAT1 phosphorylation and diminished expression of IRF9, underscoring its pivotal role in modulating the IFN-I signaling cascade. Furthermore, the NF-κB pathway, known for its integral connection with IFN-I activation and inflammatory responses, was also influenced by NFATc2 regulation. This was substantiated through rigorous validation, although further explorations are imperative to fully elucidate the underlying regulatory mechanisms. Additionally, the attenuation of cellular damage in SH-SY5Y cells co-cultured with conditioned medium post NFATc2 knockdown highlights a protective role that warrants comprehensive investigation.

Here, we analyzed scRNA-seq data together with bulk RNA-seq data, and the results improve our understanding of the complexity of PD at the single-cell level and provide several TFs that may be key regulators of IFN-I signaling under PD conditions. However, this study has several limitations. First, our analysis was limited by its small sample size. Our findings should be confirmed in larger cohorts and heterogeneous populations. Second, we validated the expression of key TFs in an MPTP/MPP^+^ model of PD neuroinflammation, but additional in vivo experiments are needed to understand how NFATc2 regulates ISG expression and disease progression.

While our current study provides valuable insights into the role of NFATc2 in the pathogenesis of PD, it also opens several avenues for further investigation. A critical aspect that warrants exploration is the identification of specific biomolecules or mechanical changes within the PD state that trigger the upregulation and activation of NFATc2, leading to the downstream effects observed in our findings. Techniques such as Co-immunoprecipitation (Co-IP) and Chromatin Immunoprecipitation sequencing (ChIP-seq) would be instrumental in identifying its cofactors and target genes in microglia, shedding light on NFATc2-regulated gene expression in PD.

Incorporating these future directions into our research agenda could significantly advance our comprehension of PD and open new therapeutic avenues. As we continue to unravel the complexities of PD, it is imperative that we explore these and other related questions to develop more effective interventions for this debilitating disease.

## Conclusion

**Fig. 10:**
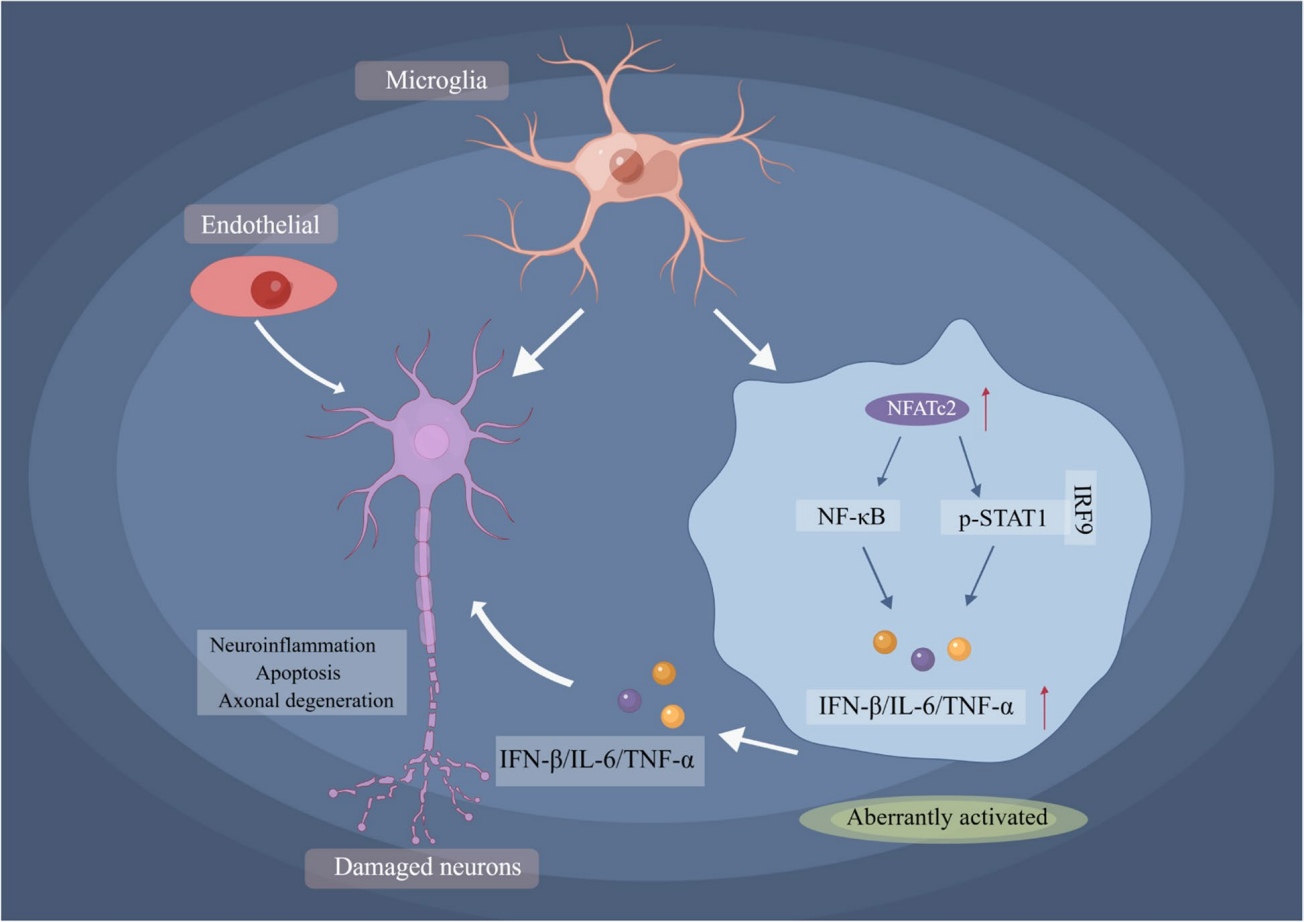
Hypothesis of possible IFN-I signaling pathway regulatory mechanism based on our findings in PD. Various toxic metabolites and proteins could increase NFATc2 expression may positively regulate expression of interferon-β and NF-κB, which in turn, enhance IFNs signaling, resulting in unexpected consequences (neuroinflammation, neuronal apoptosis, axonal degeneration, etc.). Images were drawn by Figdraw

In this study, we have characterized the heterogeneity of interferon responses across various cell clusters in Parkinson’s disease (PD) patients, employing an innovative approach integrating the AUCell algorithm with single-cell RNA sequencing (scRNA-seq). Our findings highlight microglia as the primary mediators in the IFN-I signaling pathway, suggesting that early targeting of these cells could be crucial in mitigating the cascade of activation events, potentially arresting PD progression at a nascent stage. Notably, our data reveal that the transcription factor NFATc2 plays a significant role in modulating type I interferon responses and provoking pro-inflammatory microglial activation, primarily through the activation of NF-κB and STAT1 phosphorylation pathways (Fig. 10). These insights pave the way for considering IFN-I inhibitors as a promising therapeutic avenue in the clinical management of PD.

### Supplementary Information

**Additional file 1:.** Figure S1. Quality control of scRNA-seq data. A, B The number of genes in cells and count distribution between groups before filtering (A) and after filtering (B). C The tSNE plot representing the 32 clusters across 39,024 midbrain cells from eleven individuals, including 5 PD patients and 6 healthy controls (CON). D Bar plots showing the proportion of cell types in samples. Figure S2. Pseudotime and single cell trajectory analysis by Monocle. A-C Single cell trajectory analysis for endothelial. D-F Single cell trajectory analysis for pericytes. Figure S3. Cell-type-specific regulon activity analysis of all cell types. Figure S4. The correlation of key regulons and TFs with IFN-Iscores. A Regulons concluded in M2/M3 modules in SCENIC, upregulated in PD, and upregulated in high- IFN-I-scoring microglia. B The expression levels of TFs (NFATc2, RUNX2, and NFATc2) in high- IFN-I-scoring microglia verses low- IFN-I-scoring microglia group. C-E The correlation of IFN-I scores with regulon NFATc2 (C), RUNX2 (D), and IRF5 (E). Figure S5. Quality control of bulk data. A-C PCA was applied to detect outliers in GSE7621 (A), GSE49036 (B), and GSE26927 (C). One outlier was removed in GSE7621.

**Additional file 2:.** Table S1. The siRNA sequences used in the experiments. Table S2. Information about the data sets used in this study. Table S3. 469 IFN-I-stimulated genes (ISGs) obtained in this study. Table S4. The 123 overlapped regulons involved in the regulation of NF-κB, IFN-I response, and inflammatory response pathways.

**Additional file 3:.** Table S5. In silico gene perturbation analysis results with Geneformer.

## Data Availability

For the scRNA-seq (GSE157783) and bulk RNA-seq (GSE7621, GSE49036, GSE26927), we thank the authors who generated and deposited the data. We thank National Institute of Health Gene Expression Omnibus (NIH GEO) of United States (U.S.) for storing and making available to us above datasets. The code used in the manuscript is deposited on Github (https://github.com/qneurolab/IFN-I-in-PD).
