# Supplementary material for: Single cell analysis reveals the roles and regulatory mechanisms of type-I interferons in Parkinson’s disease
Source: Cell Commun Signal. 2024 Apr 2;22:212. doi: 10.1186/s12964-024-01590-1 (PMC10985960; doi:10.1186/s12964-024-01590-1)
Supplement: Supplementary file 1 — Additional file 1:. Figure S1. Quality control of scRNA-seq data. A, B The number of genes in cells and count distribution between groups before filtering (A) and after filtering (B). C The tSNE plot representing the 32 clusters across 39,024 midbrain cells from eleven individuals, including 5 PD patients and 6 healthy controls (CON). D Bar plots showing the proportion of cell types in samples. Figure S2. Pseudotime and single cell trajectory analysis by Monocle. A-C Single cell trajectory analysis for endothelial. D-F Single cell trajectory analysis for pericytes. Figure S3. Cell-type-specific regulon activity analysis of all cell types. Figure S4. The correlation of key regulons and TFs with IFN-Iscores. A Regulons concluded in M2/M3 modules in SCENIC, upregulated in PD, and upregulated in high- IFN-I-scoring microglia. B The expression levels of TFs (NFATc2, RUNX2, and NFATc2) in high- IFN-I-scoring microglia verses low- IFN-I-scoring microglia group. C-E The correlation of IFN-I scores with regulon NFATc2 (C), RUNX2 (D), and IRF5 (E). Figure S5. Quality control of bulk data. A-C PCA was applied to detect outliers in GSE7621 (A), GSE49036 (B), and GSE26927 (C). One outlier was removed in GSE7621. [file 12964_2024_1590_MOESM1_ESM.docx]

**Supplementary Materials for**

**Single cell analysis reveals the roles and regulatory mechanisms of type-I interferons in Parkinson’s disease**

Pusheng Quan ^2,1, *^, Xueying Li ^1, *^, Yao Si ^1, *^, Linlin Sun ^1^, Fei Fan Ding ^1^, Yuwei Fan ^1^, Han Liu ^1^, Chengqun Wei ^3^, Ruihua Li ^1^, Xue Zhao ^1^, Fan Yang ^1, #^, Lifen Yao ^1, #^

^1^ Department of Neurology, The First Affiliated Hospital, Harbin Medical University, Harbin, China

^2^ Department of Neurology, The Affiliated Hospital of Inner Mongolia Medical University, Hohhot, China.

^3^ Department of General Practice, Heilongjiang Provincial Hospital, Harbin, China

^#^ Correspondence: Lifen Yao, email: [yaolf@hrbmu.edu.cn](mailto:yaolf@hrbmu.edu.cn). Fan Yang, email: [yangfan42001@163.com](mailto:yangfan42001@163.com).

^*, #^ These authors contributed equally to this work.


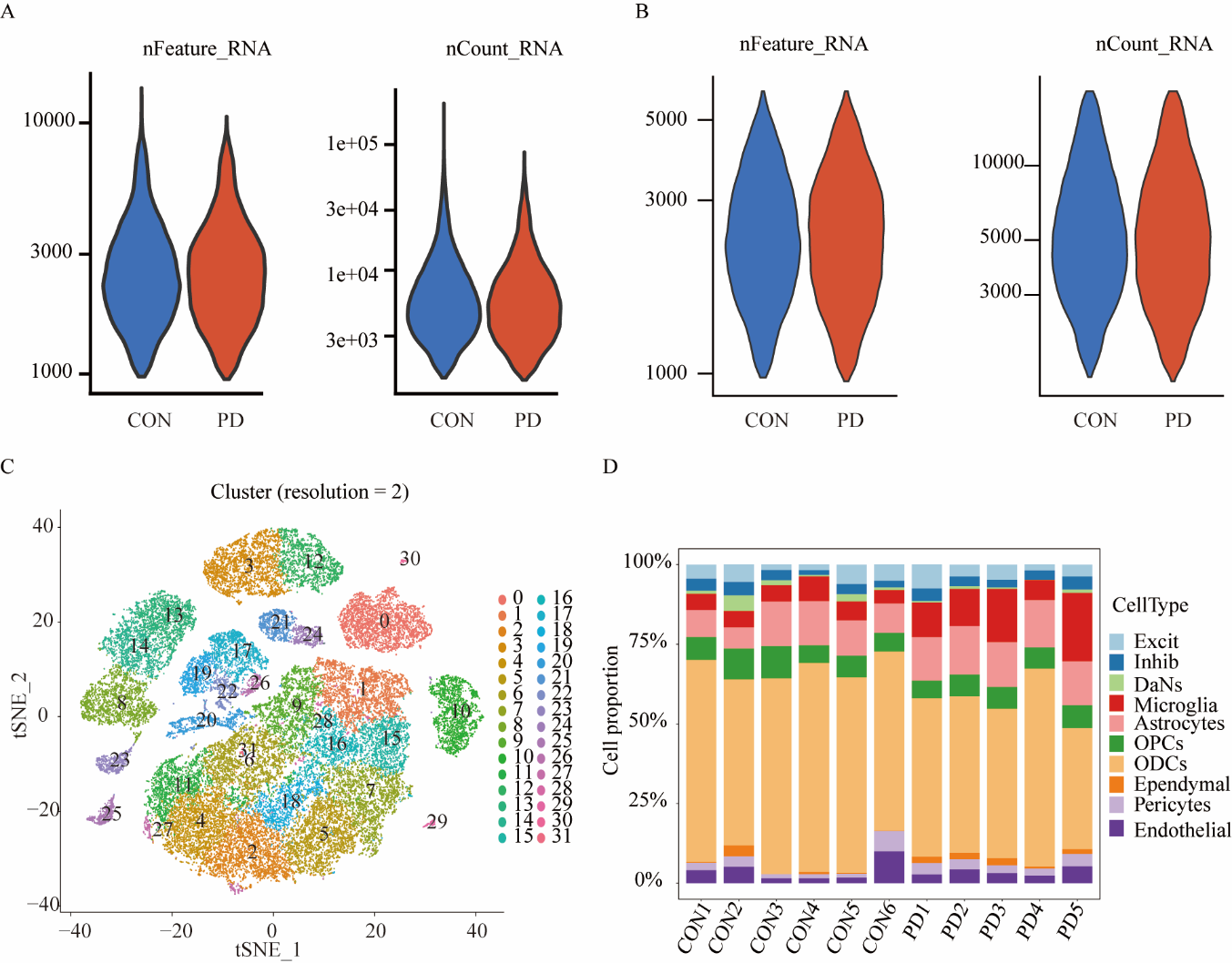


**Figure S1**. Quality control of scRNA-seq data. **A, B** The number of genes in cells and count distribution between groups before filtering (A) and after filtering (B). **C** The tSNE plot representing the 32 clusters across 39,024 midbrain cells from eleven individuals, including 5 PD patients and 6 healthy controls (CON). **D** Bar plots showing the proportion of cell types in samples.


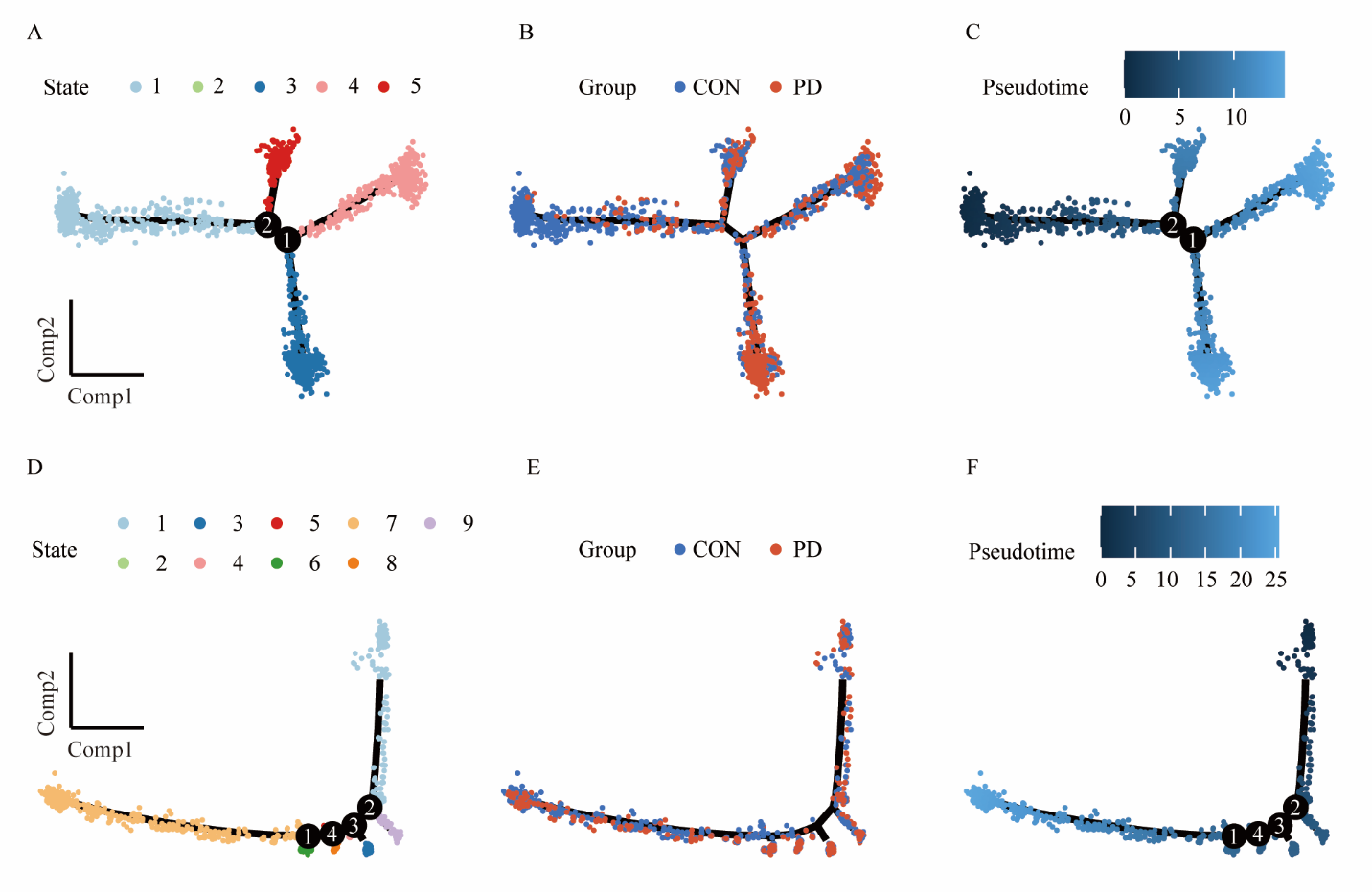


**Figure S2**. Pseudotime and single cell trajectory analysis by Monocle. **A-C** Single cell trajectory analysis for endothelial. **D-F** Single cell trajectory analysis for pericytes.


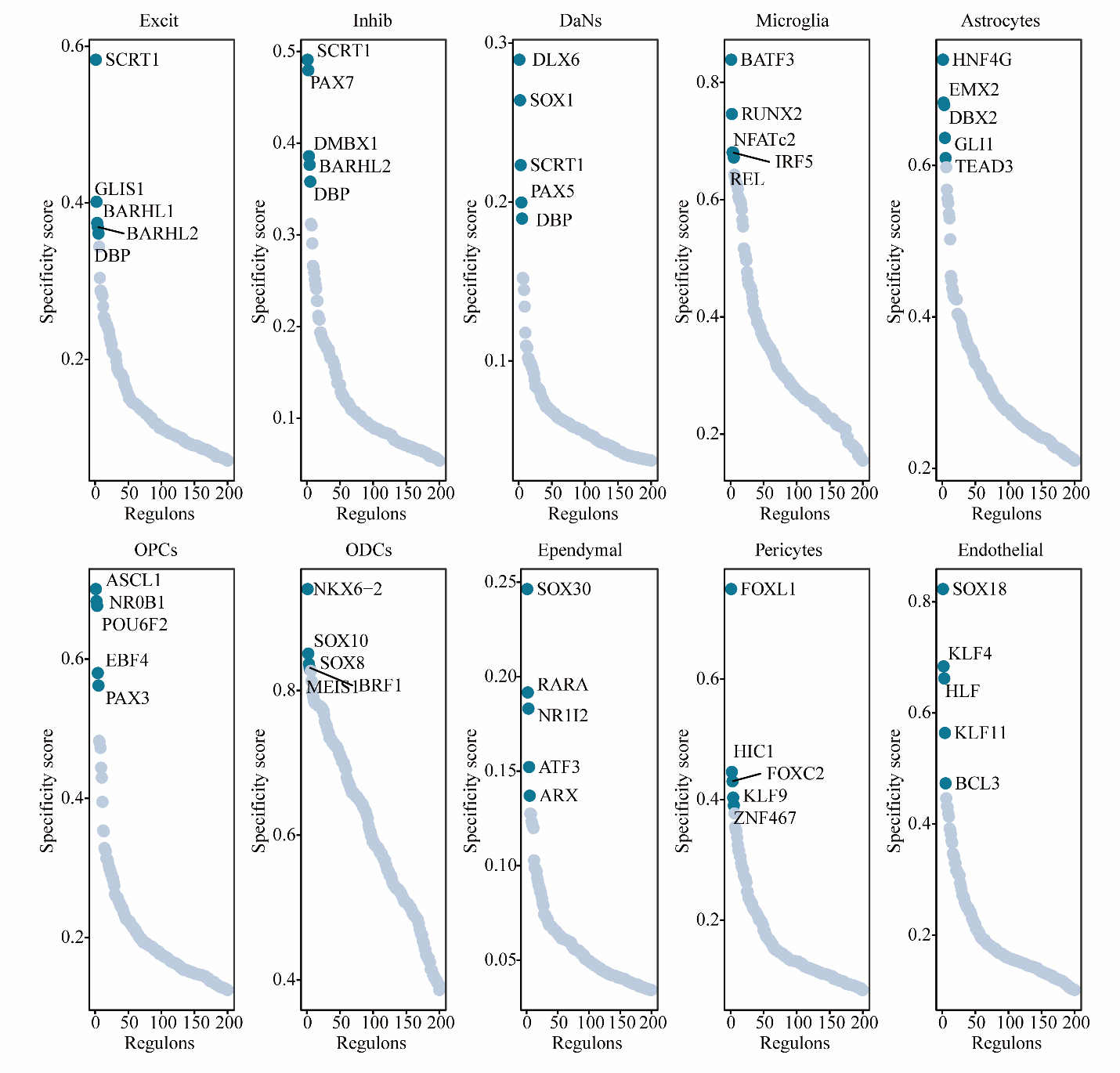


**Figure S3.** Cell-type-specific regulon activity analysis of all cell types.


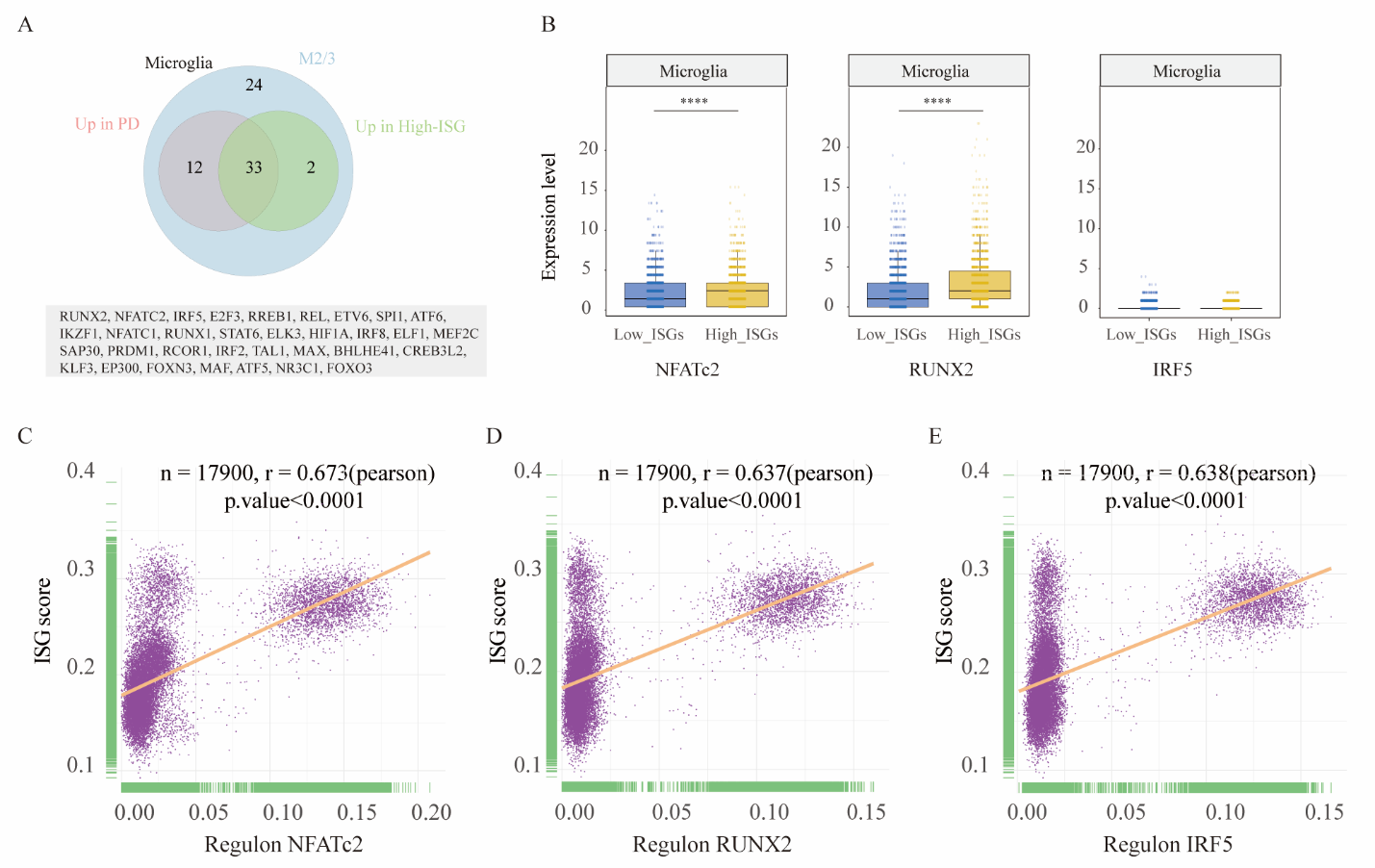


**Figure S4**. The correlation of key regulons and TFs with IFN-I scores. **A** Regulons concluded in M2/M3 modules in SCENIC, upregulated in PD, and upregulated in high- IFN-I-scoring microglia. **B** The expression levels of TFs (NFATc2, RUNX2, and NFATc2) in high- IFN-I-scoring microglia verses low- IFN-I-scoring microglia group. **C-E** The correlation of IFN-I scores with regulon NFATc2 **(C)**, RUNX2 **(D)**, and IRF5 **(E)**.


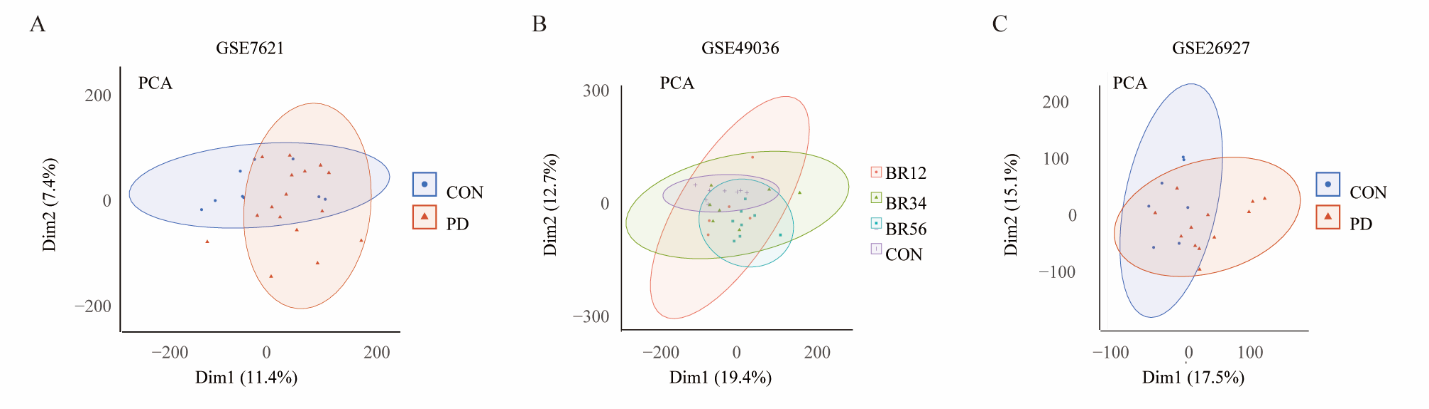


**Figure S5**. Quality control of bulk data. **A-C** PCA was applied to detect outliers in GSE7621 **(A)**, GSE49036 **(B)**, and GSE26927 **(C)**. One outlier was removed in GSE7621.
