# Supplementary material for: Single cell analysis reveals the roles and regulatory mechanisms of type-I interferons in Parkinson’s disease
Source: Cell Commun Signal. 2024 Apr 2;22:212. doi: 10.1186/s12964-024-01590-1 (PMC10985960; doi:10.1186/s12964-024-01590-1)
Supplement: Supplementary file 2 — Additional file 2:. Table S1. The siRNA sequences used in the experiments. Table S2. Information about the data sets used in this study. Table S3. 469 IFN-I-stimulated genes (ISGs) obtained in this study. Table S4. The 123 overlapped regulons involved in the regulation of NF-κB, IFN-I response, and inflammatory response pathways. [file 12964_2024_1590_MOESM2_ESM.docx]

**Table S1.** The siRNA sequences used in the experiments.

| si-NFATc2 | F | 5’-CCAGUUUGUUUACAGCUAATT-3’ |
| --- | --- | --- |
|  | R | 5’- UUAGCUGUAAACAAACUGGTT -3’ |
| Si-NC | F | 5’- UUCUCCGAACGUGUCACGUTT-3’ |
|  | R | 5’-ACGUGACACGUUCGGAGAATT-3’ |

**Table S2.** Information about the data sets used in this study.

| **Dataset** | **Platform** | **Data type** | **Source** | **Total** | **PD** | **Control** |
| --- | --- | --- | --- | --- | --- | --- |
| GSE7621 | GPL570 | Array | Substantia nigra | 24 | 15 | 9 |
| GSE49036 | GPL570 | Array | Substantia nigra | 28 | 20 | 8 |
| GSE26927 | GPL6255 | Array | Substantia nigra | 19 | 12 | 7 |
| GSE157783 | GPL24676 | scRNA-seq | Midbrain tissue | 11 | 5 | 6 |

**Table S3.** 469 IFN-I-stimulated genes (ISGs) obtained in this study.

| A2M | CADPS2 | DOK5 | GUCY1A2 | MAN1A1 | PDZD2 | SAMSN1 | SULF2 |
| --- | --- | --- | --- | --- | --- | --- | --- |
| ABCC4 | CAMK1D | DPYSL5 | GYG2 | MAN2A1 | PECAM1 | SAT1 | SYN2 |
| ABCG2 | CAMK2G | EDIL3 | HCK | MAOB | PELI1 | SCD | SYNE2 |
| ABHD2 | CCDC146 | EDN1 | HES4 | MAP2 | PEX5L | SCIN | SYNJ2 |
| ACOT11 | CCDC40 | EFNA5 | HIF1A | MAP3K1 | PFKFB3 | SCN9A | SYT17 |
| ACSBG1 | CCSER1 | ELOVL7 | HIF3A | MAP3K5 | PFKP | SDC4 | TBC1D4 |
| ACSL1 | CD34 | EMP2 | HIGD1B | MARCKS | PHACTR2 | SEC14L1 | TBXAS1 |
| ACSL5 | CD36 | ENG | HIP1 | MBP | PHLDA1 | SEMA3C | TCF7L2 |
| ADAM28 | CD44 | ENPP2 | HIVEP1 | MECOM | PID1 | SERPINE2 | TENM2 |
| ADAMTS9 | CD74 | EPAS1 | HLA-A | MEF2C | PIK3AP1 | SEZ6L | TF |
| AGBL1 | CD86 | EPB41L4A | HLA-B | MERTK | PIK3R5 | SGCD | TFEC |
| AK8 | CD99 | EPSTI1 | HLA-C | MFSD2A | PITPNC1 | SGK1 | TFPI |
| AKAP12 | CDH18 | ERC2 | HLA-DRA | MGAT4A | PLA2G4A | SGPP2 | TGFB2 |
| AKAP13 | CDH23 | ESRRG | HLA-DRB1 | MGLL | PLCB1 | SH3GL3 | TGFBR1 |
| ALOX5 | CDK18 | ESYT2 | HLA-E | MGST1 | PLCE1 | SIDT1 | TGFBR2 |
| ALPK1 | CEBPD | ETS2 | HSPA1A | MOBP | PLOD2 | SKAP2 | TIMP3 |
| AMPH | CELF2 | ETV6 | HSPA1B | MS4A6A | PLSCR4 | SLA | TLR1 |
| ANGPT2 | CERS6 | EXPH5 | HSPB1 | MSR1 | PLXDC2 | SLC11A1 | TLR2 |
| ANK3 | CFH | EXT1 | ID1 | MT1E | PLXNA2 | SLC16A1 | TM4SF1 |
| ANO2 | CHL1 | EZR | ID3 | MT1M | PODXL | SLC19A1 | TM6SF1 |
| ANTXR2 | CHST15 | FAM107A | IFI16 | MT1X | PON2 | SLC1A1 | TMEM144 |
| ANXA1 | CHST9 | FAM13A | IFI27 | MT2A | PPFIBP1 | SLC1A2 | TMEM163 |
| ANXA3 | CHSY1 | FAR2 | IFITM2 | MTHFD1L | PPM1L | SLC24A4 | TMTC1 |
| APBB1IP | CLMN | FBXO32 | IFITM3 | MYL9 | PRELP | SLC25A37 | TNC |
| APCDD1 | CMTM8 | FCHSD2 | IGSF21 | MYO10 | PRICKLE1 | SLC2A1 | TNFRSF1B |
| APLP1 | CMYA5 | FGD2 | IL16 | MYO1F | PRKAG2 | SLC2A13 | TNR |
| APOE | CNDP1 | FGF2 | IL18 | MYOF | PRKG1 | SLC2A3 | TOX |
| APOLD1 | CNP | FGFR2 | IL4R | NAMPT | PRR16 | SLC2A5 | TPM1 |
| ARHGAP10 | CNTNAP5 | FGFR3 | INPP5D | NCALD | PRUNE2 | SLC30A1 | TRPM2 |
| ARHGAP25 | COBLL1 | FLRT2 | ITGA2 | NDRG2 | PTPRD | SLC30A10 | TTLL7 |
| ARHGAP26 | COL27A1 | FLT1 | ITGAX | NEBL | PTPRE | SLC39A10 | TTTY14 |
| ARHGAP29 | COL4A2 | FN1 | ITPKB | NEDD9 | PTPRO | SLC4A4 | TXNIP |
| ARHGAP42 | COL8A1 | FNDC3B | ITPR1 | NET1 | QDPR | SLC7A1 | UNC5C |
| ARHGEF10L | COLEC12 | FOS | ITPR2 | NFIB | RANBP3L | SLC8A1 | USP6NL |
| ARL15 | CPE | FOSL2 | JAG1 | NHS | RASSF9 | SLCO4A1 | UTRN |
| ARPP21 | CPED1 | FRMD3 | JUN | NOTCH3 | RBM47 | SLIT3 | VAMP5 |
| ASIC2 | CPM | FRMD4A | KALRN | NR2F2 | RBMS1 | SMAD6 | VCAN |
| ATP10A | CPNE8 | FRMD6 | KCND3 | NR3C2 | RBMS2 | SNRPN | VRK2 |
| ATP1B2 | CRIM1 | FRY | KCNH8 | NRCAM | RBMS3 | SNTB1 | VWF |
| ATP2B2 | CRYAB | FUT9 | KCNMA1 | NRG1 | RCSD1 | SORBS1 | WDFY4 |
| ATP8B4 | CSF1R | GABBR2 | KCNQ3 | NRXN3 | REL | SORBS2 | XAF1 |
| AXL | CST3 | GAD2 | KCTD12 | NSG2 | RGS17 | SOX9 | ZBTB46 |
| B2M | CTSB | GAP43 | KIAA1217 | NTRK2 | RHBDF2 | SP100 | ZFHX3 |
| BAALC | CTSC | GBE1 | KIAA1671 | NTRK3 | RHOC | SPAG6 | ZFP36L2 |
| BACH1 | CUX2 | GGT5 | KLF12 | OGFRL1 | RIMKLB | SPARC | ZFPM2 |
| BCL6 | DAB1 | GIMAP7 | KLHL29 | OSBPL11 | RIN3 | SPOCK2 | ZNF365 |
| BGN | DAB2IP | GJA4 | KSR2 | P2RY12 | RNF144B | SPP1 | ZNF366 |
| BLNK | DAPK1 | GLDN | LAPTM5 | P2RY14 | RNF19A | SPRED2 | ZNF516 |
| BMPR1B | DCLK2 | GLIS3 | LAT2 | PAG1 | RNF220 | SPTAN1 | ZNF710 |
| BSG | DENND1B | GMDS | LGALS1 | PALD1 | ROBO2 | SPTBN4 |  |
| C1QA | DENND3 | GMPR | LIMS1 | PALLD | ROR2 | SRGAP2 |  |
| C3 | DGKG | GNB4 | LMO2 | PAM | RORA | SRGAP2B |  |
| C5AR1 | DIAPH2 | GPCPD1 | LPAR1 | PAPSS2 | RPGR | SRGAP2C |  |
| C8orf34 | DLGAP2 | GPRIN3 | LPCAT2 | PARD3B | RREB1 | ST18 |  |
| CABLES1 | DNAAF1 | GRIK2 | LPL | PARP14 | RUNX1T1 | ST6GAL1 |  |
| CACNA1A | DNAH14 | GRIN2A | LRIG1 | PARP8 | RUNX2 | ST6GALNAC3 | |
| CACNA1B | DNAH7 | GRIN3A | LRP1 | PCSK5 | S100A6 | ST8SIA4 |  |
| CACNA1C | DOCK2 | GRIP2 | LRRFIP1 | PCSK6 | S100B | STIM1 |  |
| CACNA1D | DOCK5 | GRK5 | LY6E | PDE1C | SAMD4A | STOM |  |
| CADPS | DOCK8 | GRM1 | LY86 | PDE3B | SAMHD1 | STON2 |  |

**Table S4.** 123 overlapped regulons obtained in this study.

| ETV6(1975g) | RELA(737g) | ELF1(3310g) | ZNF821(399g) | NRF1(1792g) |
| --- | --- | --- | --- | --- |
| SPI1(1454g) | ATF5(65g) | SOX8(795g) | E2F6(273g) | SMARCA4(2401g) |
| IKZF1(1265g) | CEBPB(596g) | EGR1(203g) | ZNF274(278g) | KDM5A(2321g) |
| IRF8(1062g) | RUNX3(254g) | ZEB1(2297g) | PHF8(490g) |  |
| PRDM1(484g) | NFATC1(313g) | SIN3A(643g) | SUPT20H(658g) |  |
| ELK3(986g) | CEBPA(133g) | TBX15(562g) | MITF(1194g) |  |
| REL(365g) | MAFF(23g) | SRF(217g) | TAF1(1332g) |  |
| STAT3(808g) | RREB1(1088g) | ATF6(491g) | MAZ(844g) |  |
| ETS2(1154g) | CD59(10g) | TEAD3(57g) | NR3C1(1543g) |  |
| RELB(229g) | NFIL3(315g) | GABPB1(536g) | HCFC1(426g) |  |
| RUNX1(1028g) | ATF3(795g) | EP300(1196g) | ELF2(2737g) |  |
| IRF5(537g) | IRF2(318g) | KLF11(296g) | NR2C2(1012g) |  |
| FLI1(1909g) | XBP1(271g) | GABPA(501g) | JUND(863g) |  |
| IRF1(764g) | HIF1A(758g) | ZIC2(411g) | CTCF(744g) |  |
| BCL3(295g) | IRF7(120g) | NFYA(316g) | TFDP1(572g) |  |
| ERG(1165g) | NR2F2(763g) | PBX3(218g) | USF2(778g) |  |
| STAT6(474g) | JUN(241g) | RFX2(2582g) | MEIS1(1104g) |  |
| CEBPD(883g) | KLF6(787g) | NFYC(369g) | RFX3(3416g) |  |
| NFKB2(171g) | KLF2(784g) | RFX4(993g) | SREBF2(1929g) |  |
| TAL1(320g) | E2F3(499g) | BACH2(136g) | SUZ12(793g) |  |
| ELF4(216g) | SOX10(638g) | ETV5(503g) | XRCC4(819g) |  |
| STAT1(863g) | ELK4(739g) | FOXO1(176g) | RAD21(2215g) |  |
| ETV7(173g) | GATA2(602g) | EZH2(185g) | BCLAF1(1582g) |  |
| RUNX2(323g) | FOXO3(294g) | E2F4(332g) | CHD2(625g) |  |
| HIVEP1(486g) | SIX1(235g) | HDAC2(461g) | CREB1(1164g) |  |
| NFATC2(235g) | RCOR1(551g) | ZMIZ1(1961g) | TAF7(1336g) |  |
| MEF2C(277g) | SREBF1(1391g) | FOXP2(1061g) | BRF1(1486g) |  |
| ETS1(456g) | SAP30(328g) | POLR2A(1176g) | KDM5B(1327g) |  |
| MAF(344g) | BHLHE41(676g) | ZNF143(514g) | MXI1(2975g) |  |
| JUNB(278g) | KLF4(113g) | MAX(1849g) | YY1(2337g) |  |
